# Supplementary material for: Antiparasitic Constituents of Beilschmiedia louisii and Beilschmiedia obscura and Some Semisynthetic Derivatives (Lauraceae)
Source: Molecules. 2020 Jun 21;25(12):2862. doi: 10.3390/molecules25122862 (PMC7357032; doi:10.3390/molecules25122862)
Supplement: Supplementary file 1 [file molecules-25-02862-s001.pdf]

Article

Antiparasitic Constituents of *Beilschmiedia Louisii* and *Beilschmiedia Obscura* and Some Semisynthetic Derivatives (Lauraceae)

Christine C. Waleguele [1], Brice M. Mba'ning [1], Angelbert F. Awantu [2], Jean J. K. Bankeu [2], Yannick S. F. Fongang [3,*], Augustin S. Ngouela [1], Etienne Tsamo [1], Norbert Sewald [4], Bruno N. Lenta [5,*] and Rui W. M. Krause [6]

[1] Department of Organic Chemistry, Faculty of Science, University of Yaoundé I, P.O. Box 812 Yaoundé, Cameroon; wwaleguele@yahoo.fr (C.C.W.); brice_mbaning@yahoo.fr (B.M.M.); sngouela@yahoo.fr (A.S.N.); tsamoet@yahoo.fr (E.T.)

[2] Department of Chemistry, Faculty of Science, The University of Bamenda, P.O. Box 39 Bambili, Cameroon; aawantu@gmail.com (A.F.A.); bankeu@gmail.com (J.J.K.B.)

[3] Department of Chemistry, Higher Teacher Training College, The University of Maroua, P.O. Box 55 Maroua, Cameroon

[4] Department of Chemistry, Bielefeld University, P.O. Box 100131, 33501 Bielefeld, Germany; norbert.sewald@uni-bielefeld.de

[5] Department of Chemistry, Higher Teacher Training College, University of Yaoundé I, P.O. Box 47 Yaoundé, Cameroon

[6] Department of Chemistry, Rhodes University, P.O. Box 94 Grahamstown 6140, South Africa; r.krause@ru.ac.za

* Correspondence: fongangfys@yahoo.fr (Y.S.F.F.); lentabruno@yahoo.fr (B.N.L.); Tel.: +237 696 13 28 33 (Y.S.F.F.); +237 675 09 75 61 (B.N.L.)

Received: 20 May 2020; Accepted: 18 June 2020; Published: 21 June 2020

Table of Contents

Figure S1: ^{13}C NMR ($CDCl_3$, 100 MHz) spectrum of compound **11a** 3
Figure S2: ^{1}H-NMR ($CDCl_3$, 400 MHz) spectrum of compound **11a** 5
Figure S3: ^{13}C DEPT-135 ($CDCl_3$, 100 MHz) spectrum of compound **11a** 6
Figure S4: COSY ($CDCl_3$, 400 MHz) spectrum of compound **11a** 6
Figure S5: HSQC ($CDCl_3$, 400 MHz) spectrum of compound **11a** 7
Figure S6: HMBC ($CDCl_3$, 400 MHz) spectrum of compound **11a** 7
Figure S7: NOESY ($CDCl_3$, 400 MHz) spectrum of compound **11a** 8
Figure S8: ^{13}C NMR ($CDCl_3$, 100 MHz) spectrum of compound **11b** 8
Figure S9: ^{1}H-NMR ($CDCl_3$, 400 MHz) spectrum of compound **11b** 9
Figure S10: ESI mass spectrum of compound **11c** 10
Figure S11:^{1}H NMR (Pyridine-d_5, 400 MHz) spectrum of compound **11c** 11
Figure S12: ^{13}C-NMR (Pyridine-d_5, 100 MHz) spectrum of compound **11c** 12
Figure S13: COSY (Pyridine-d_5, 400 MHz) spectrum of compound **11c** 12
Figure S14: HSQC (Pyridine-d_5, 400 MHz) spectrum of compound **11c** 13
Figure S15: HMBC (Pyridine-d_5, 400 MHz) spectrum of compound **11c** 13
Figure S16: NOESY (Pyridine-d_5, 400 MHz) spectrum of compound **11c** 14
Figure S17: ^{1}H-NMR ($CDCl_3$, 400 MHz) spectrum of compound **11d** 15
Figure S18: ^{13}C-NMR ($CDCl_3$, 100 MHz) spectrum of compound **11d** 16
Figure S19: HSQC ($CDCl_3$, 400 MHz) spectrum of compound **11d** 17
Figure S20: ESI MASS spectrum of the mixture of compound **1** and **2** 18
Figure S21: ^{1}H-NMR ($CDCl_3$, 600 MHz) spectrum of the mixture of compound **1** and **2** 20
Figure S22:^{13}C-NMR ($CDCl_3$, 150 MHz) spectrum of the mixture of compound **1** and **2** 21
Figure S23: COSY ($CDCl_3$, 600 MHz) spectrum of the mixture of compound **1** and **2** 22
Figure S24: HSQC ($CDCl_3$, 600 MHz) spectrum of the mixture of compound **1** and **2** 23
Figure S25: HMBC ($CDCl_3$, 600 MHz) spectrum of the mixture of compound **1** and **2** 25
Figure S26: ^{1}H-NMR ($CDCl_3$, 400 MHz) spectrum of compound **3** 26
Figure S27: ^{13}C-NMR ($CDCl_3$, 100 MHz) spectrum of compound **3** 27
Figure S28: COSY ($CDCl_3$, 400 MHz) spectrum of compound **3** 28
Figure S29: HSQC ($CDCl_3$, 400 MHz) spectrum of compound **3** 28
Figure S30: HMBC ($CDCl_3$, 400 MHz) spectrum of compound **3** 29
Figure S31: ^{13}C-NMR ($CDCl_3$, 150 MHz) spectrum of compound **4** 30
Figure S32: ^{1}H-NMR ($CDCl_3$, 600 MHz) spectrum of compound **4** 30
Figure S33: ^{1}H-NMR ($CDCl_3$, 300 MHz) spectrum of compound **11** 31
Figure S34: ^{13}C-NMR ($CDCl_3$, 75 MHz) spectrum of compound **11** 32

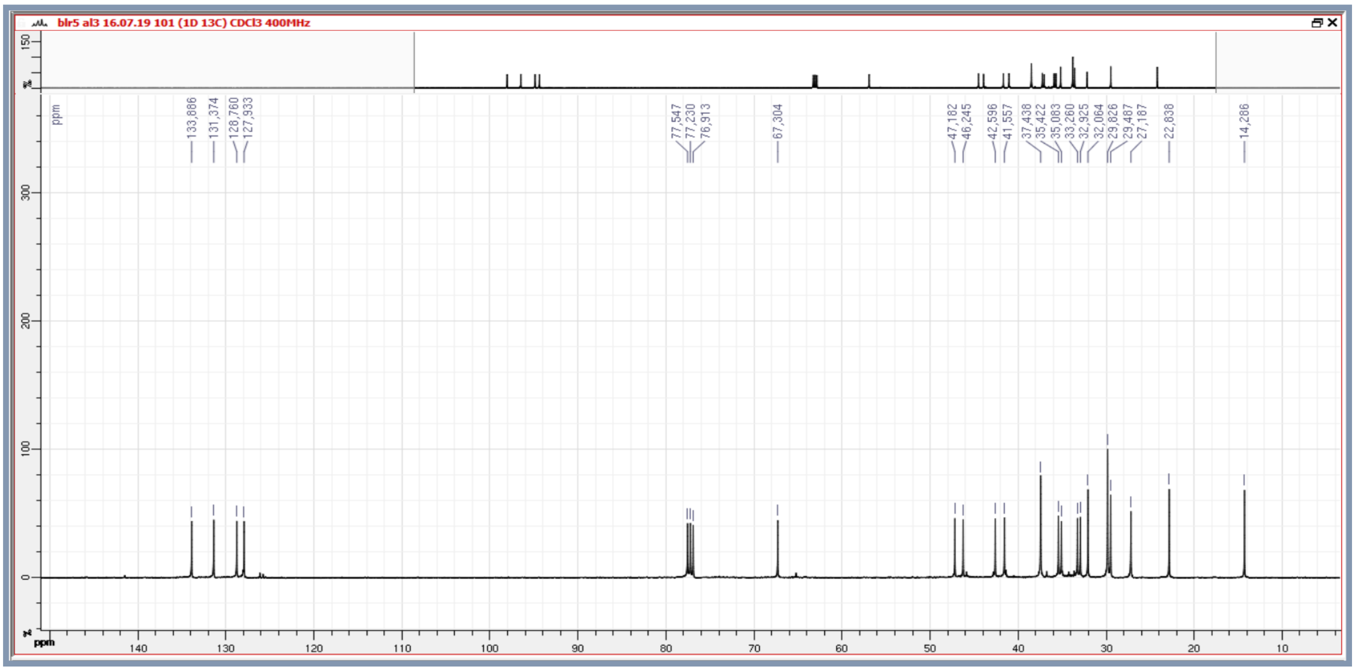

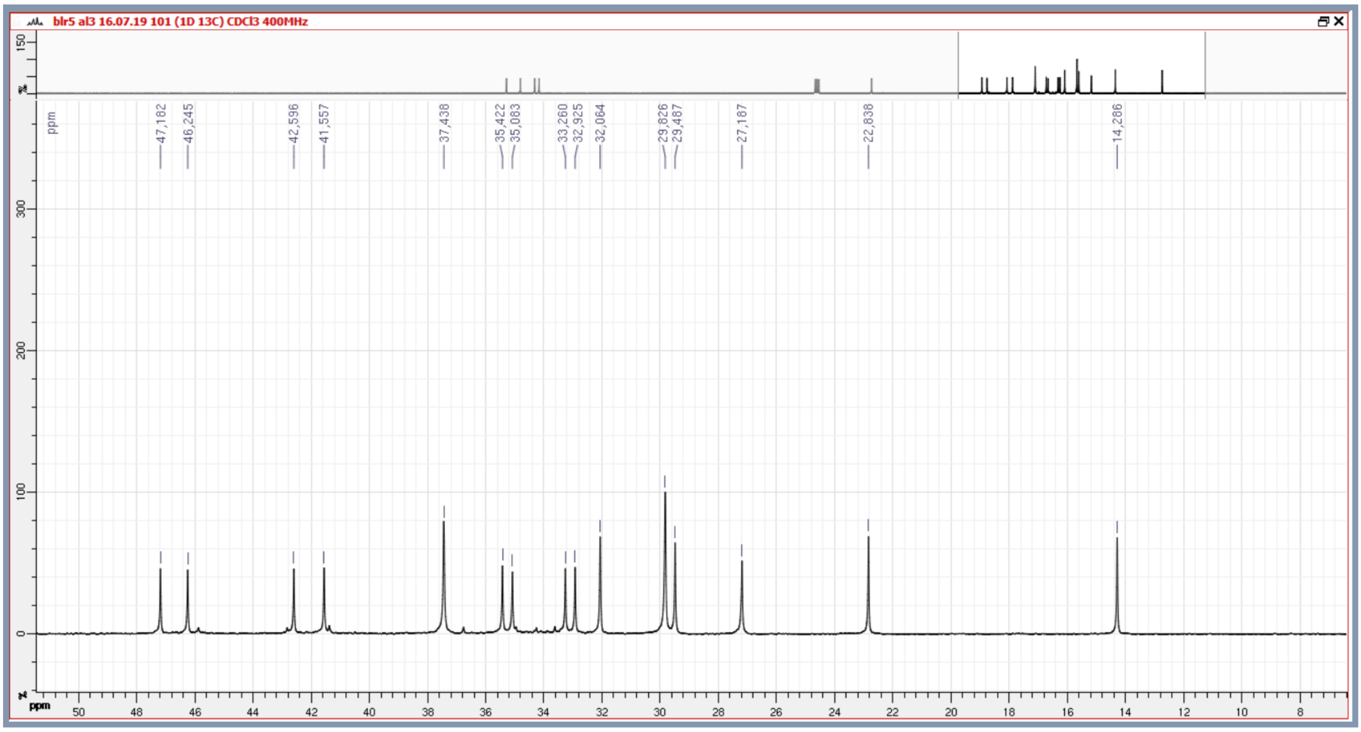

Figure S1: ^{13}C NMR ($CDCl_3$, 100 MHz) spectrum of compound **11a**

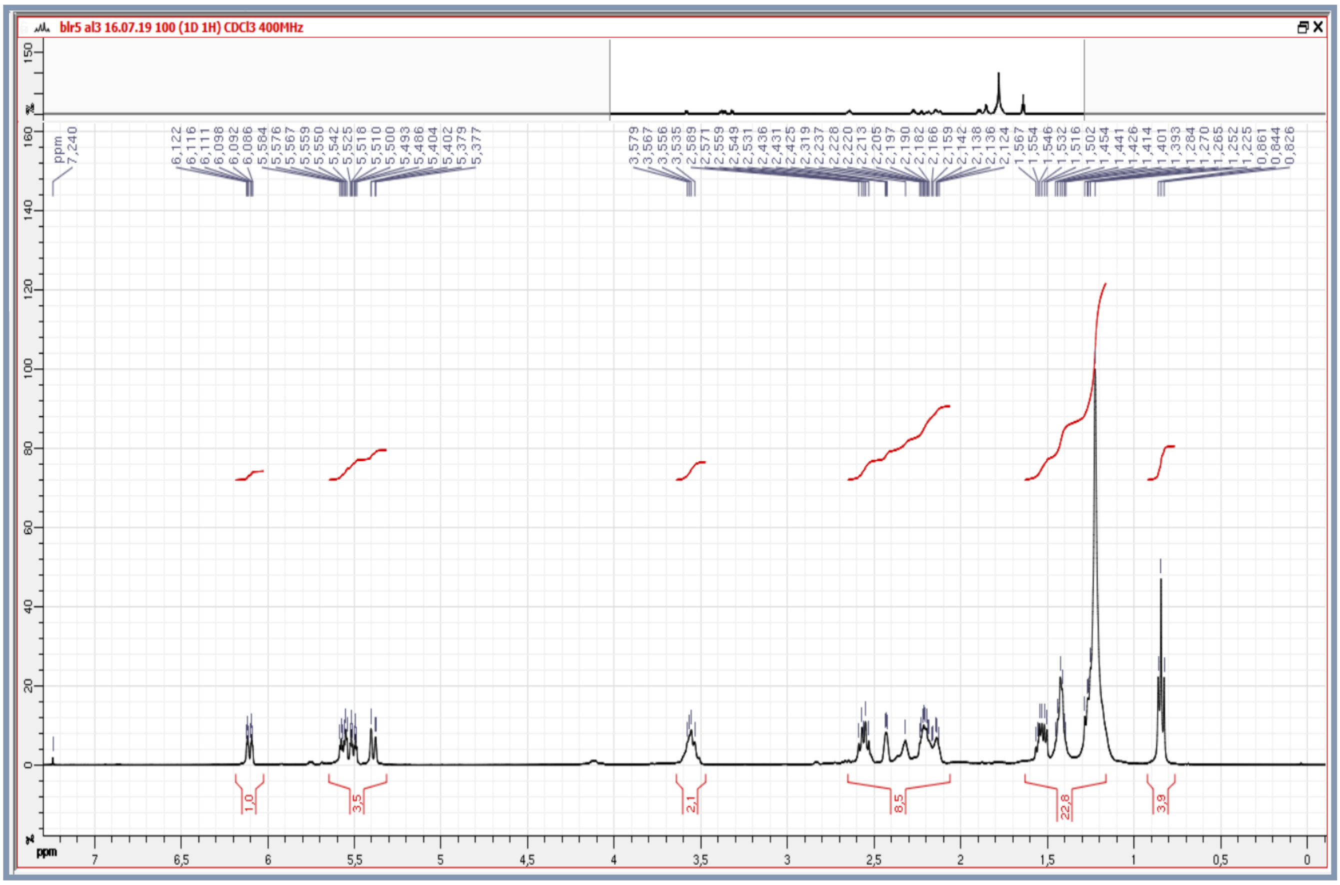
blr5 al3 16.07.19 100 (1D 1H) CDCl3 400MHz

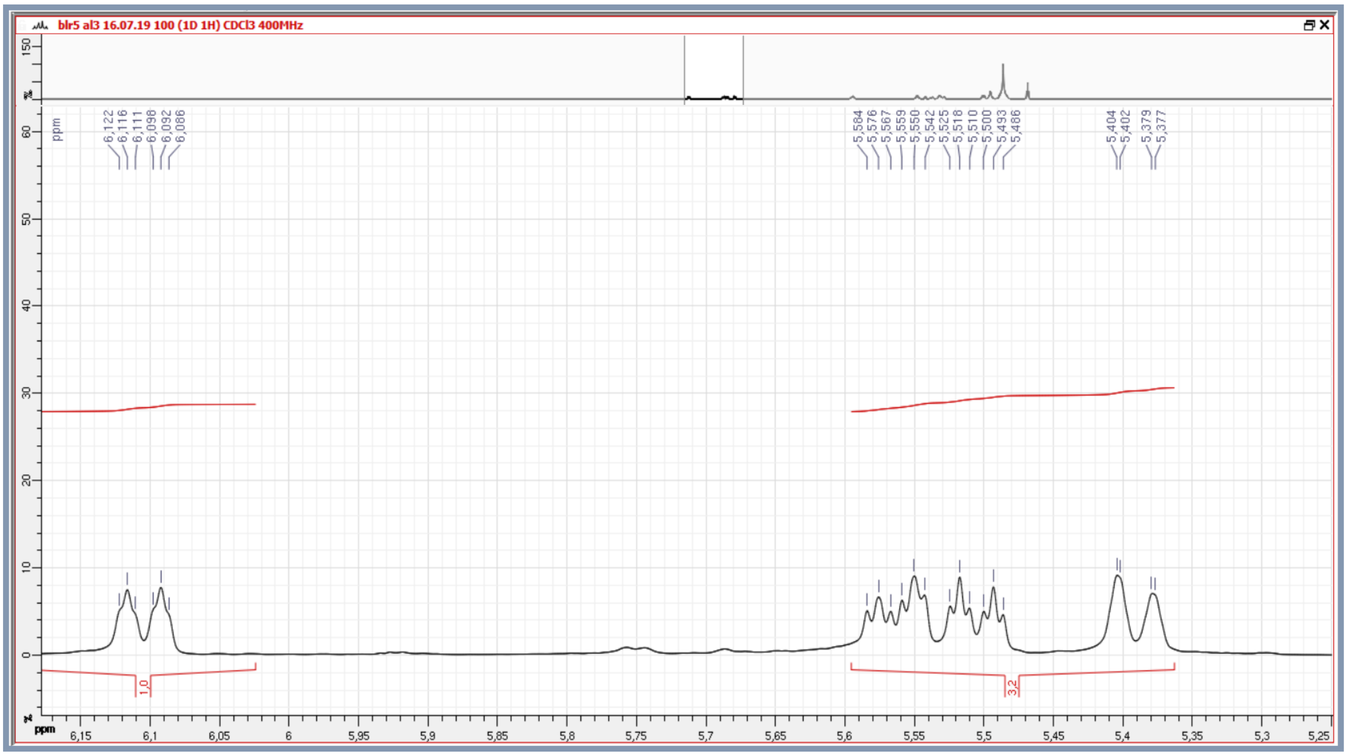
blr5 al3 16.07.19 100 (1D 1H) CDCl3 400MHz

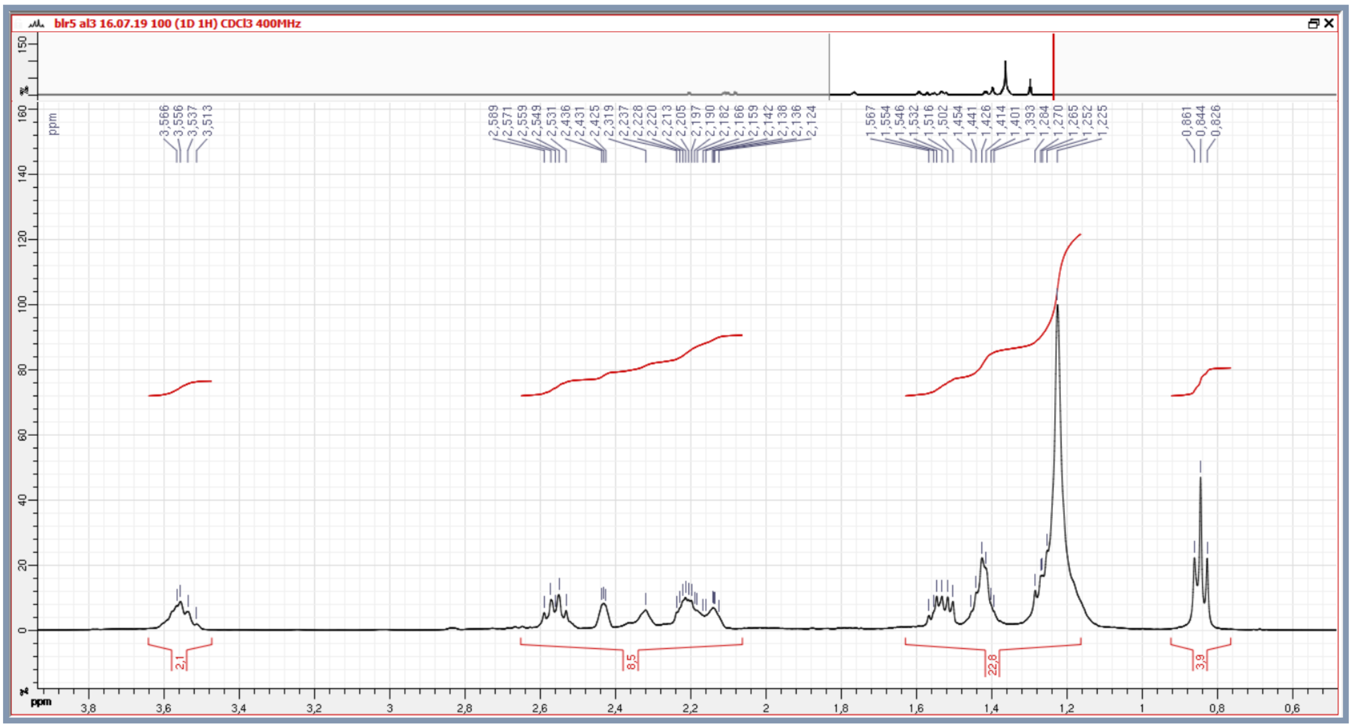

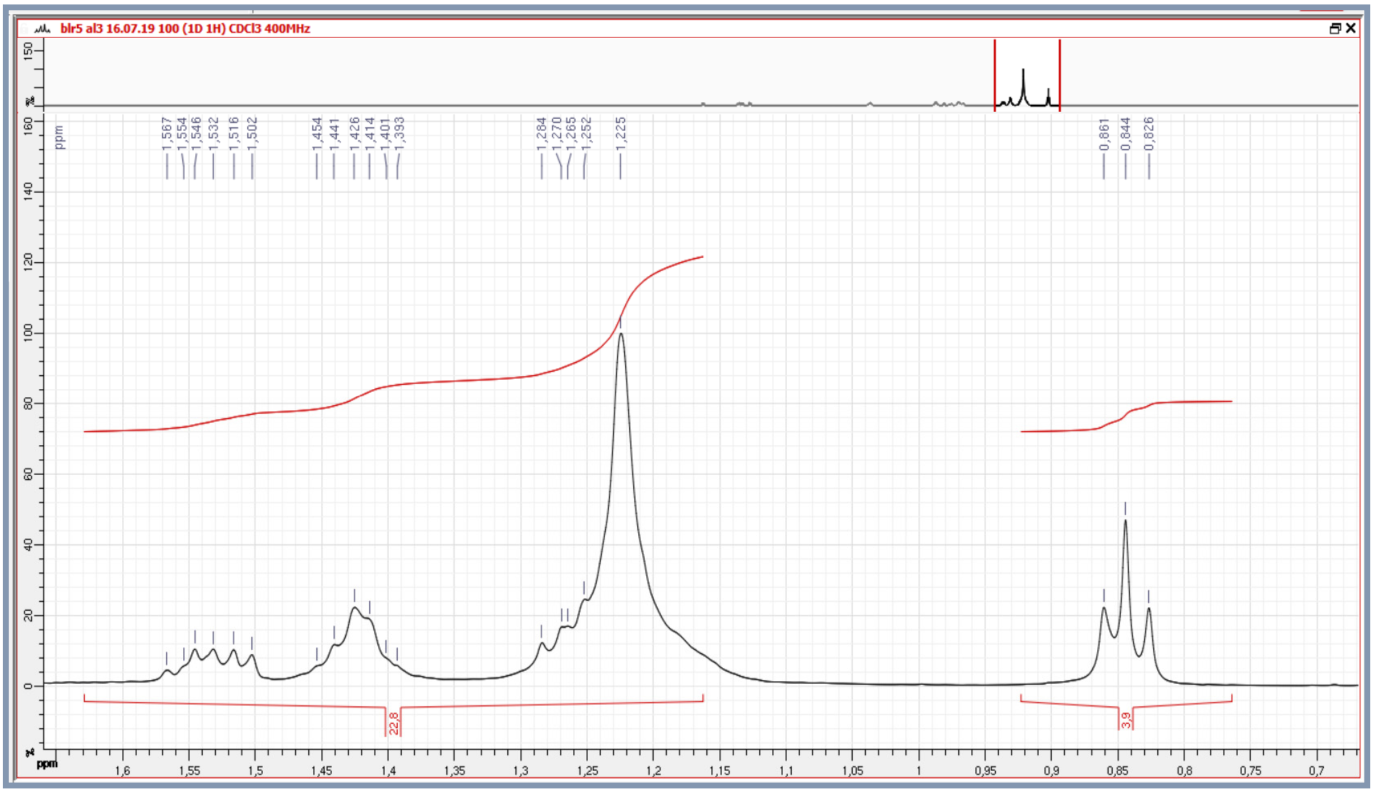

Figure S2: ^{1}H-NMR ($CDCl_3$, 400 MHz) spectrum of compound **11a**

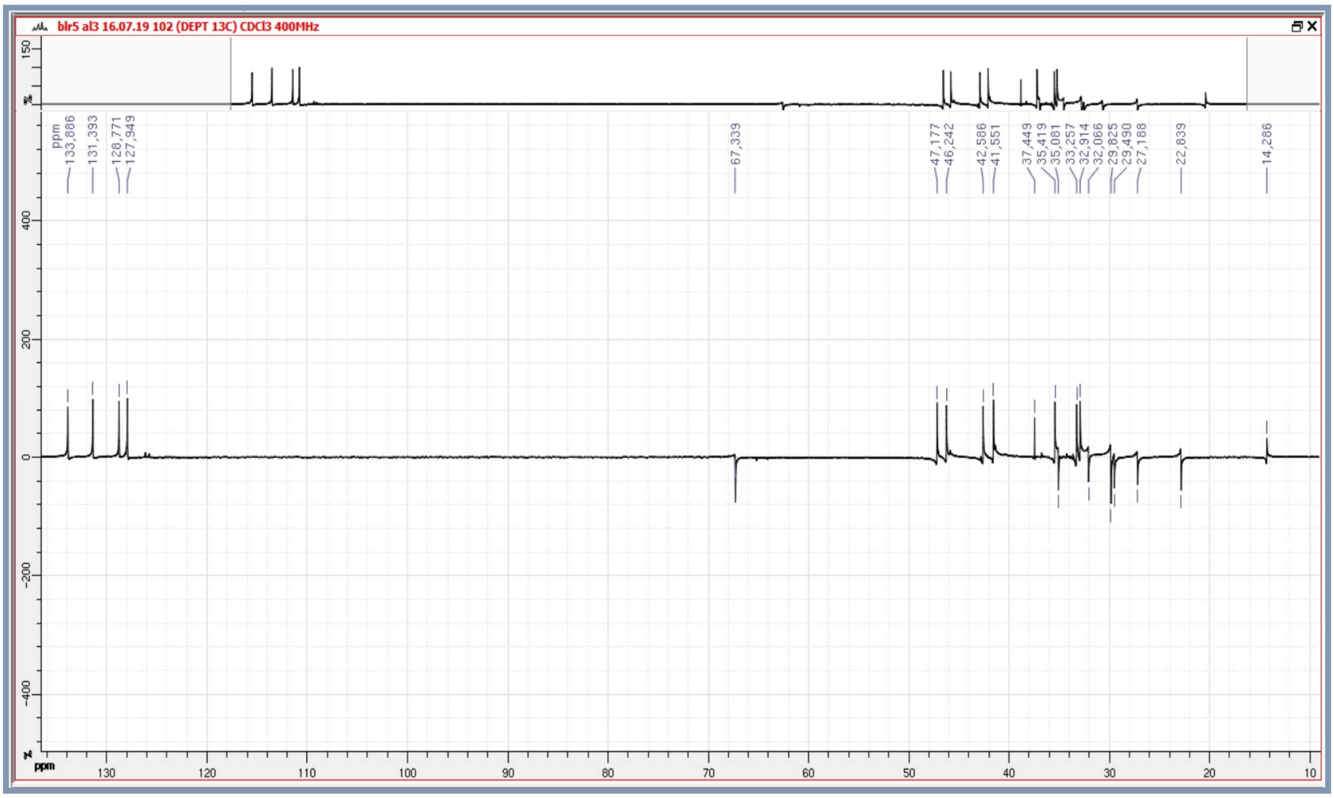

Figure S3: ^{13}C DEPT-135 ($CDCl_3$, 100 MHz) spectrum of compound **11a**

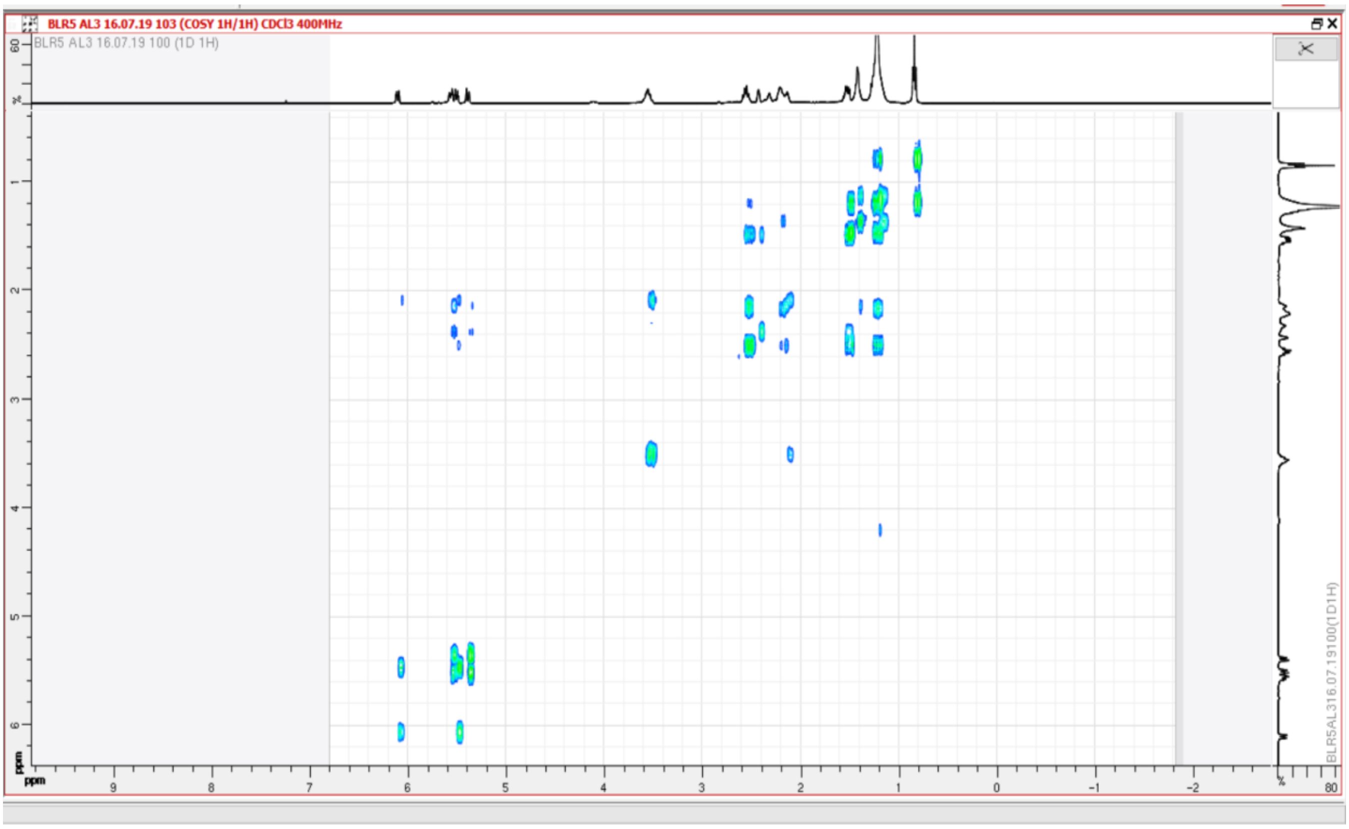

Figure S4: COSY ($CDCl_3$, 400 MHz) spectrum of compound **11a**

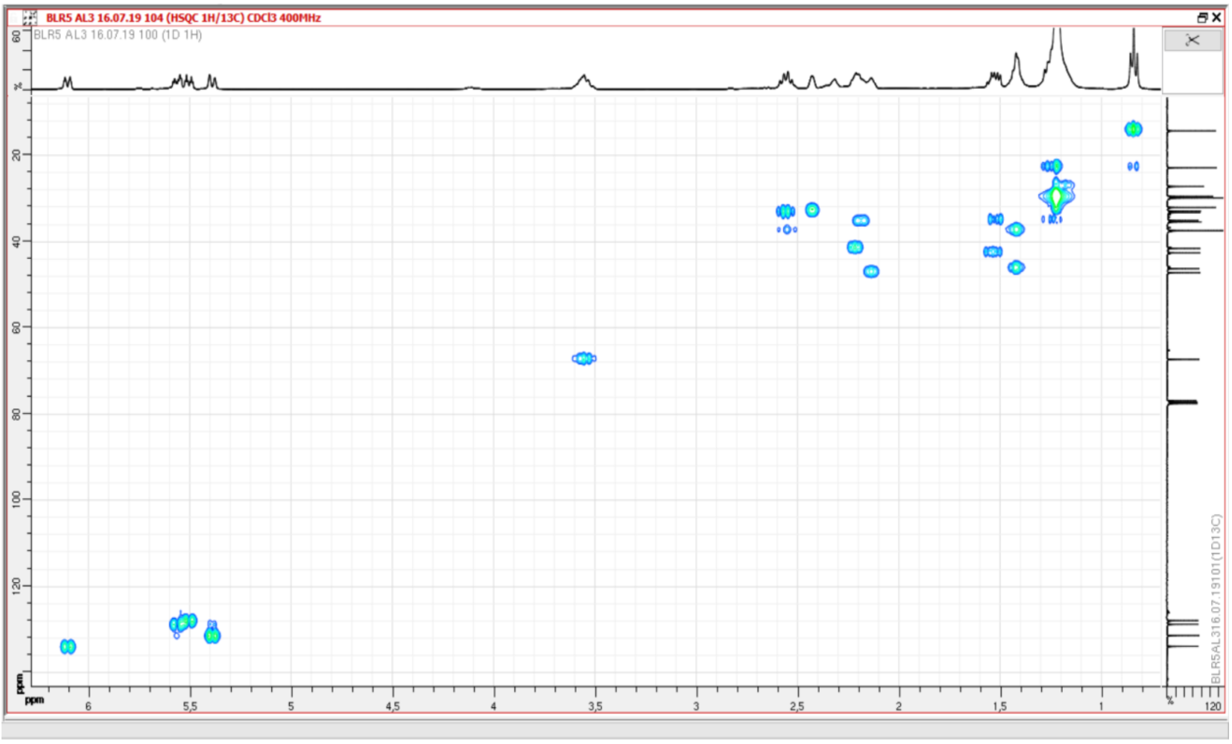

Figure S5: HSQC ($CDCl_3$, 400 MHz) spectrum of compound **11a**

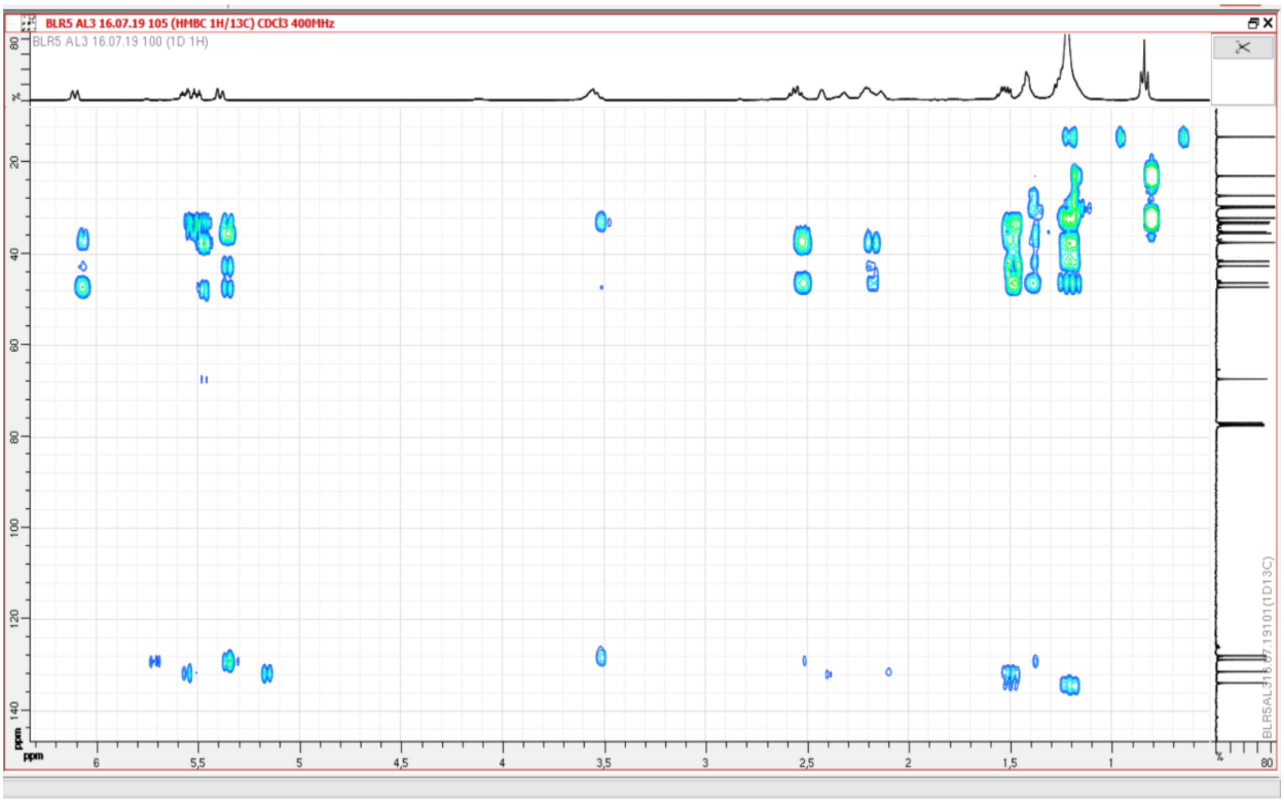

Figure S6: HMBC ($CDCl_3$, 400 MHz) spectrum of compound **11a**

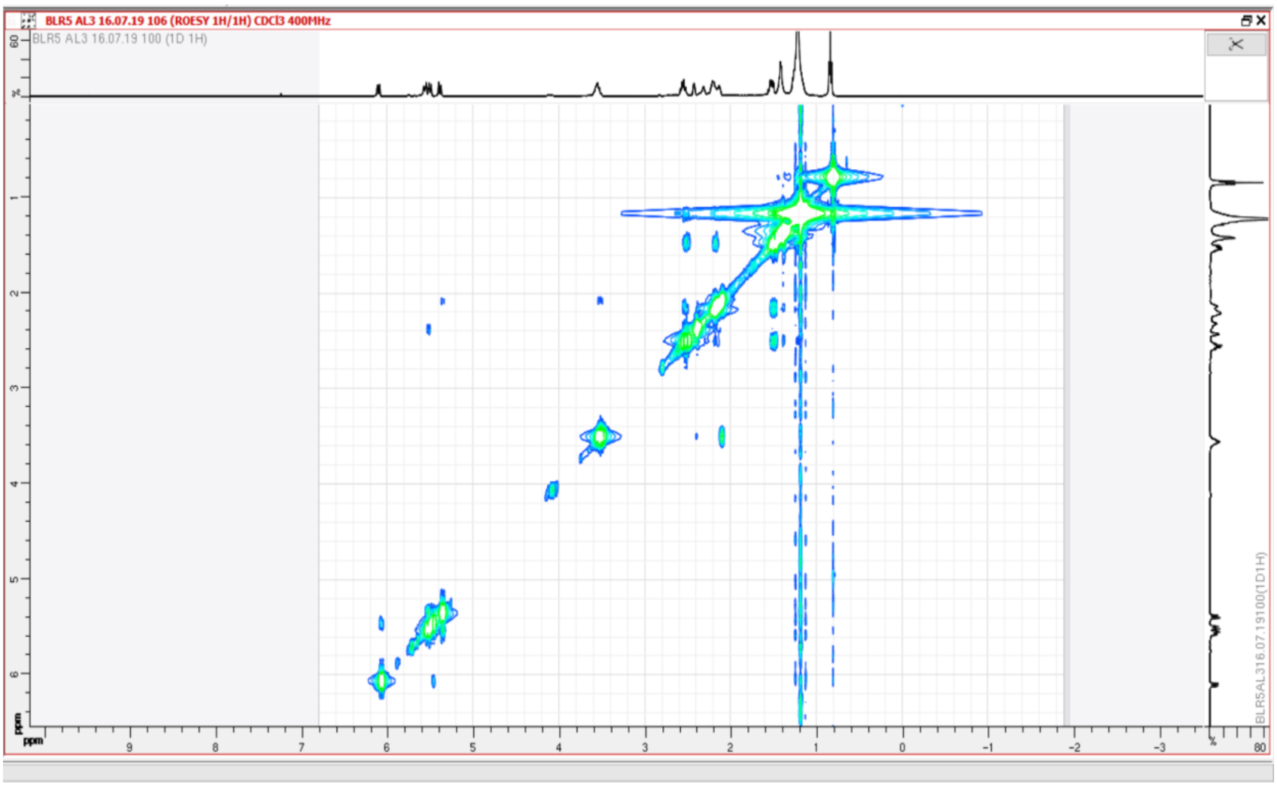

Figure S7: NOESY ($CDCl_3$, 400 MHz) spectrum of compound **11a**

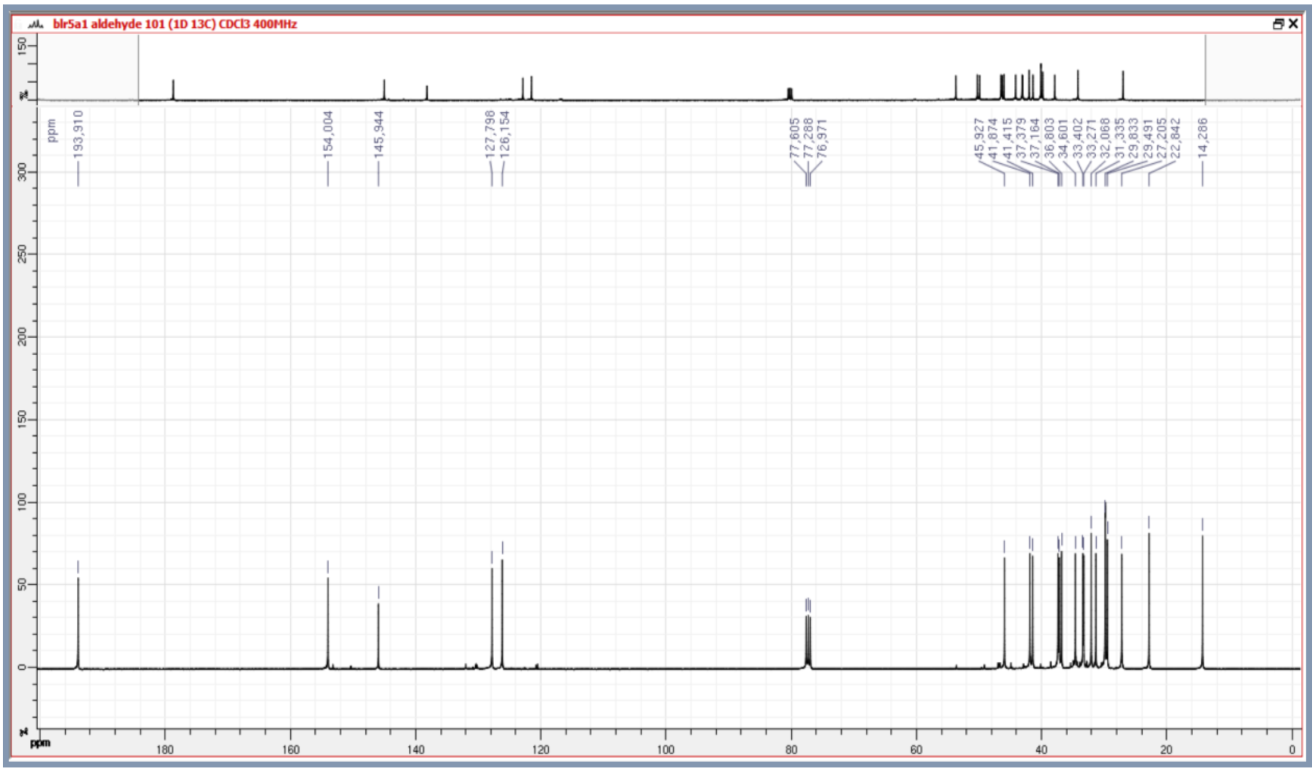

Figure S8: ^{13}C NMR ($CDCl_3$, 100 MHz) spectrum of compound **11b**

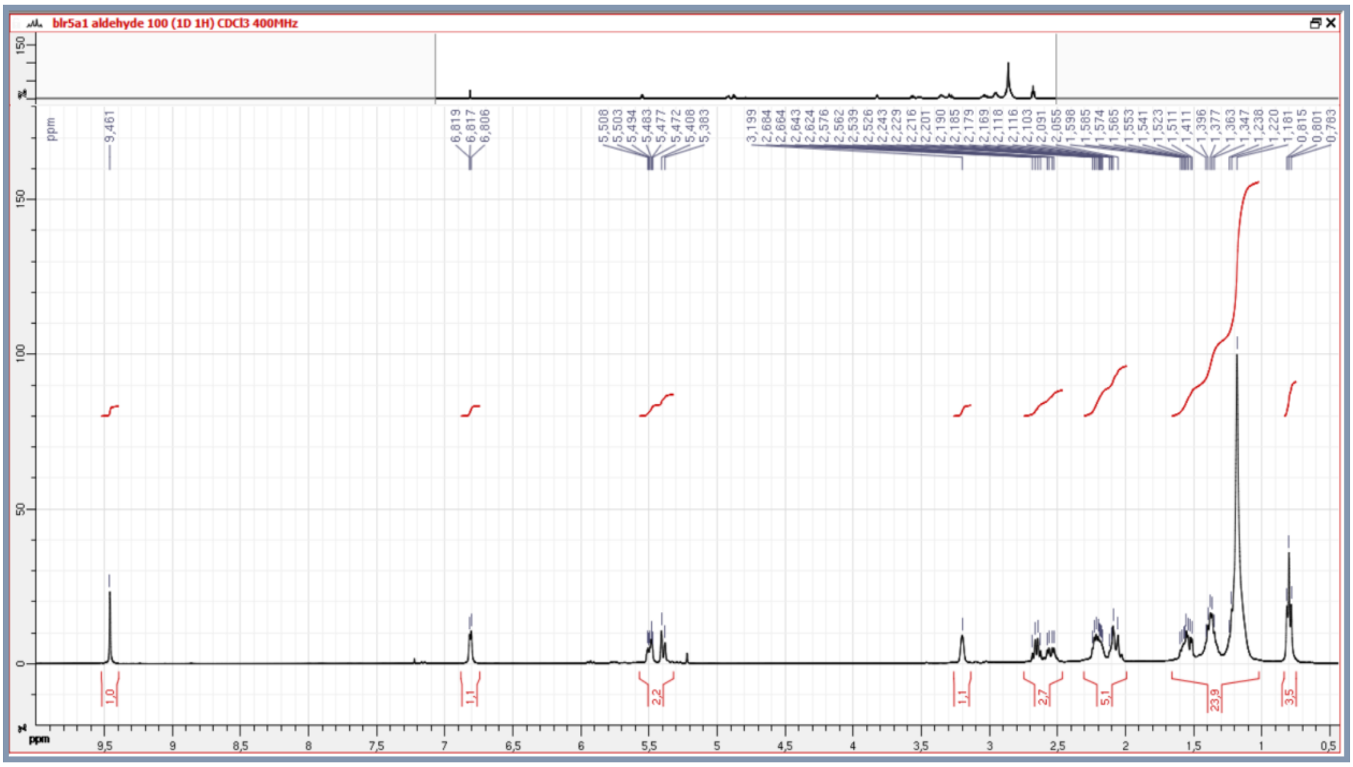

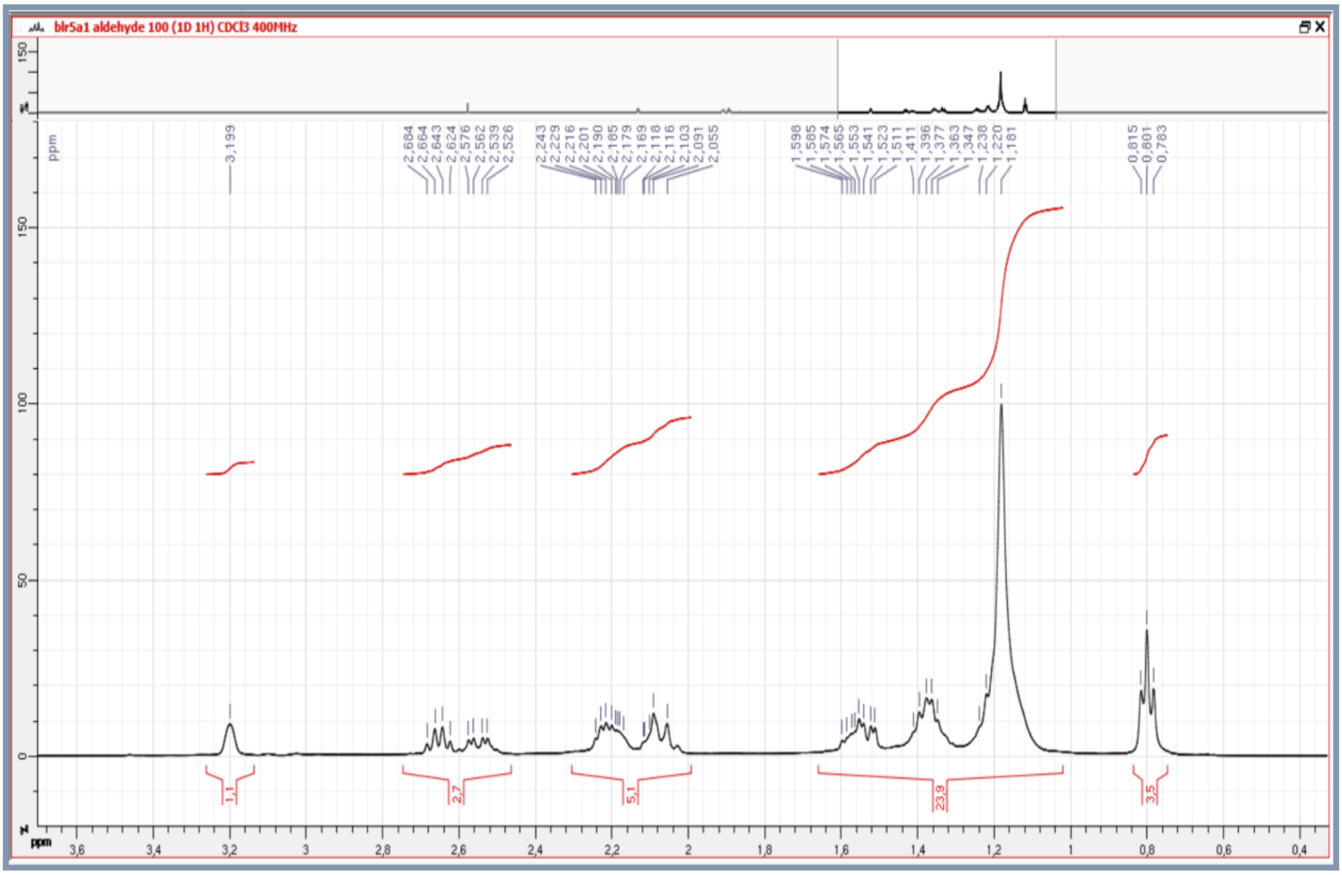

Figure S9: ^{1}H-NMR ($CDCl_3$, 400 MHz) spectrum of compound **11b**

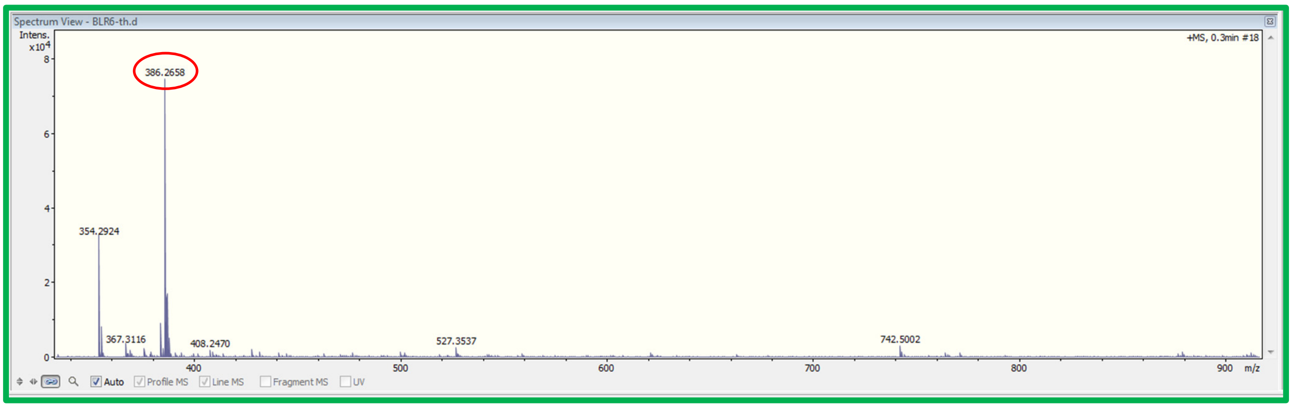

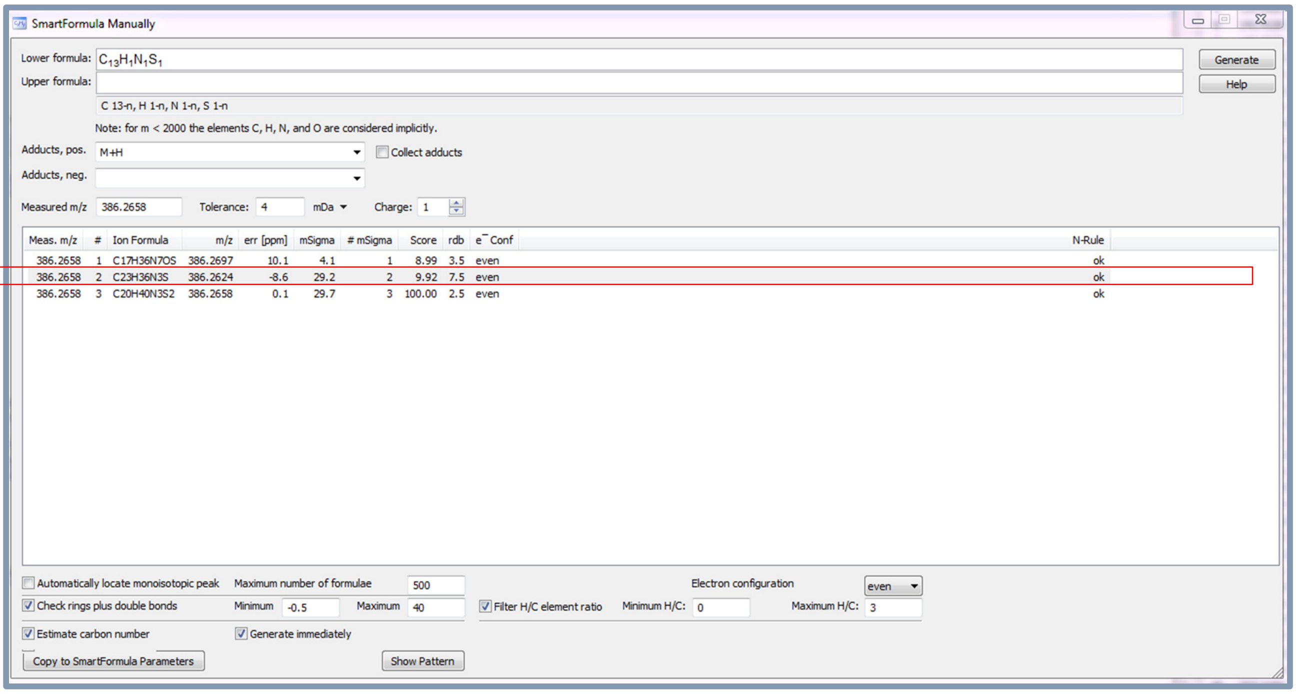

Figure S10: ESI mass spectrum of compound **11c**

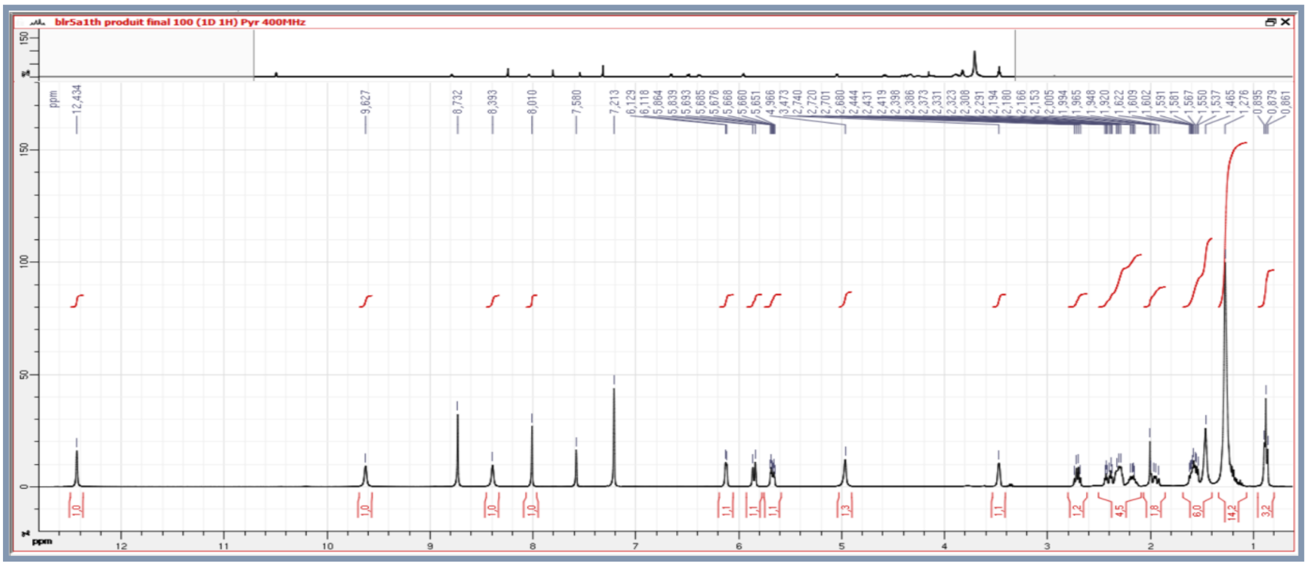

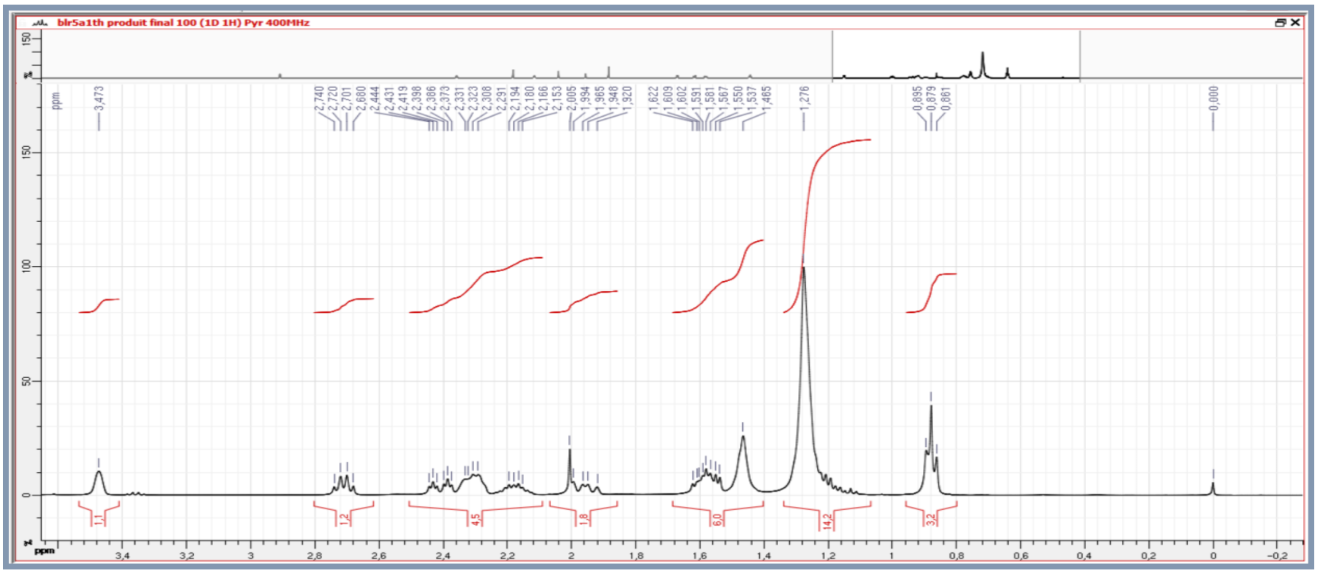

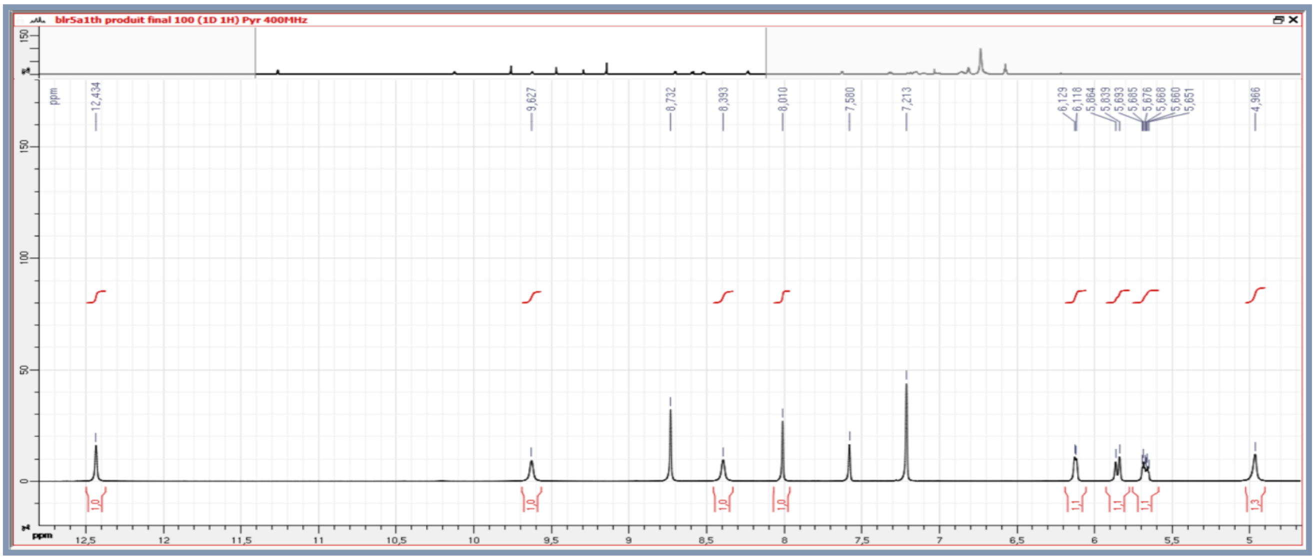

Figure S11:^{1}H NMR (Pyridine-d_5, 400 MHz) spectrum of compound **11c**

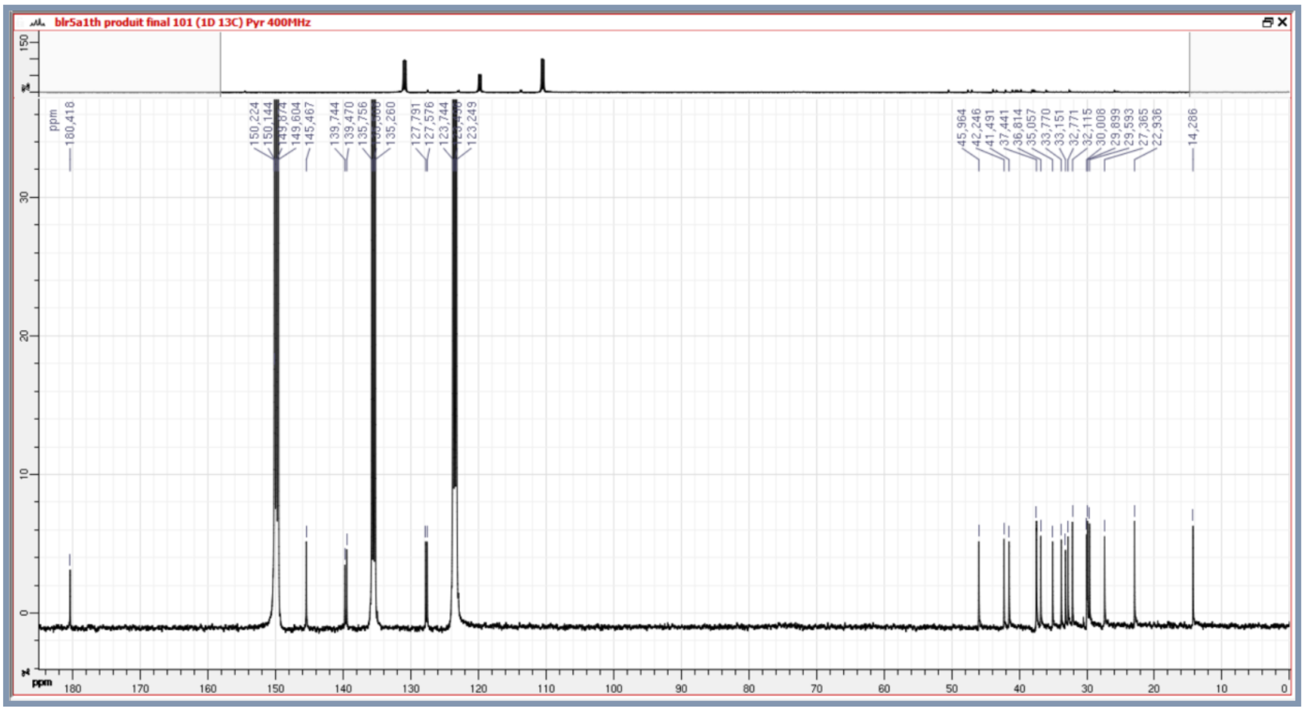

Figure S12: ^{13}C-NMR (Pyridine-d_5, 100 MHz) spectrum of compound **11c**

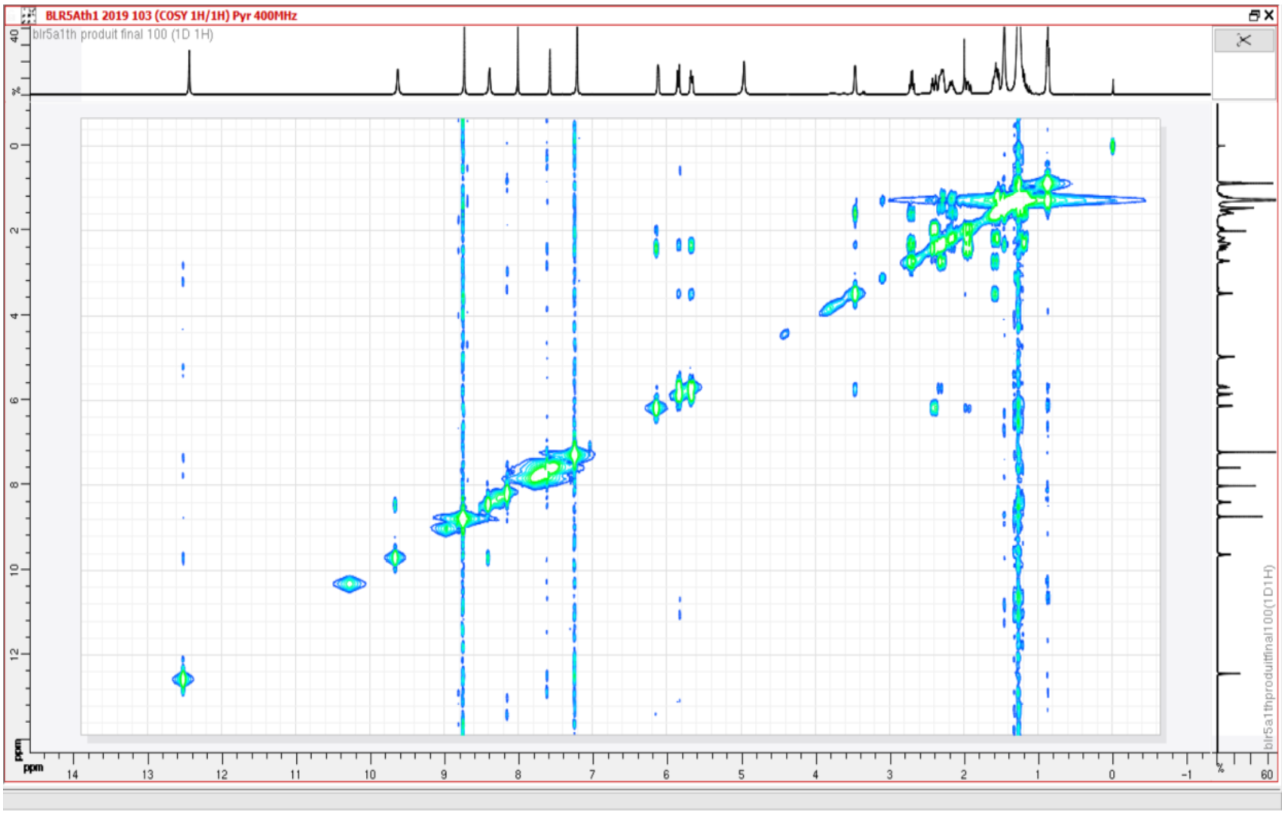

Figure S13: COSY (Pyridine-d_5, 400 MHz) spectrum of compound **11c**

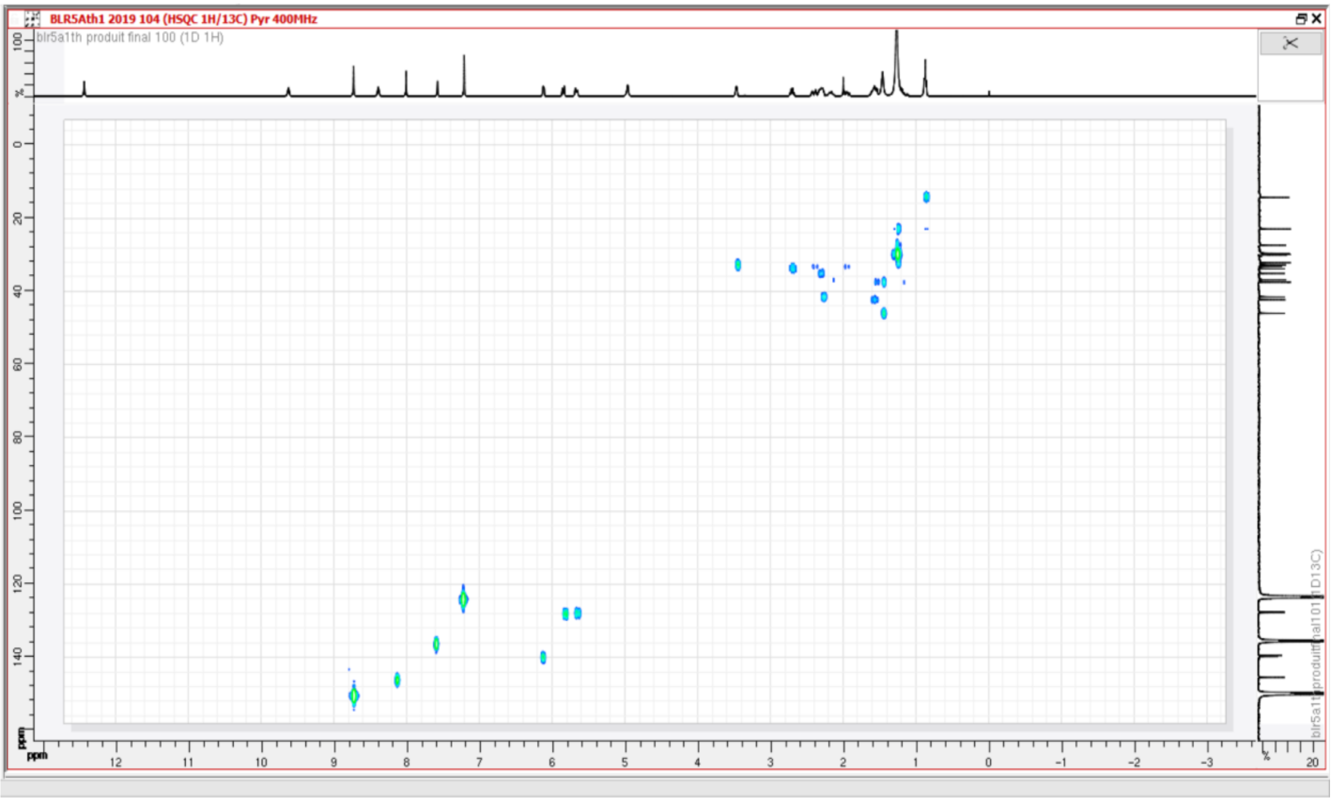

Figure S14: HSQC (Pyridine-d_5, 400 MHz) spectrum of compound **11c**

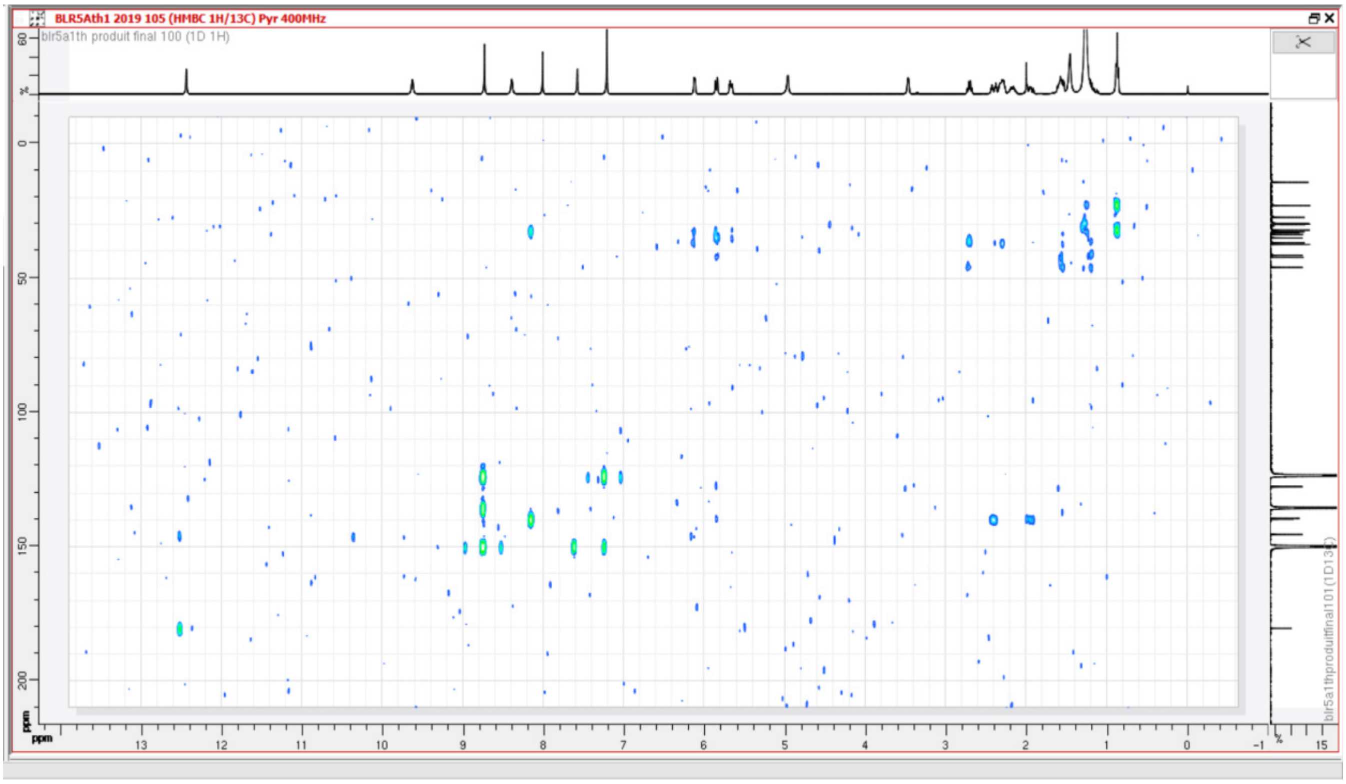

Figure S15: HMBC (Pyridine-d_5, 400 MHz) spectrum of compound **11c**

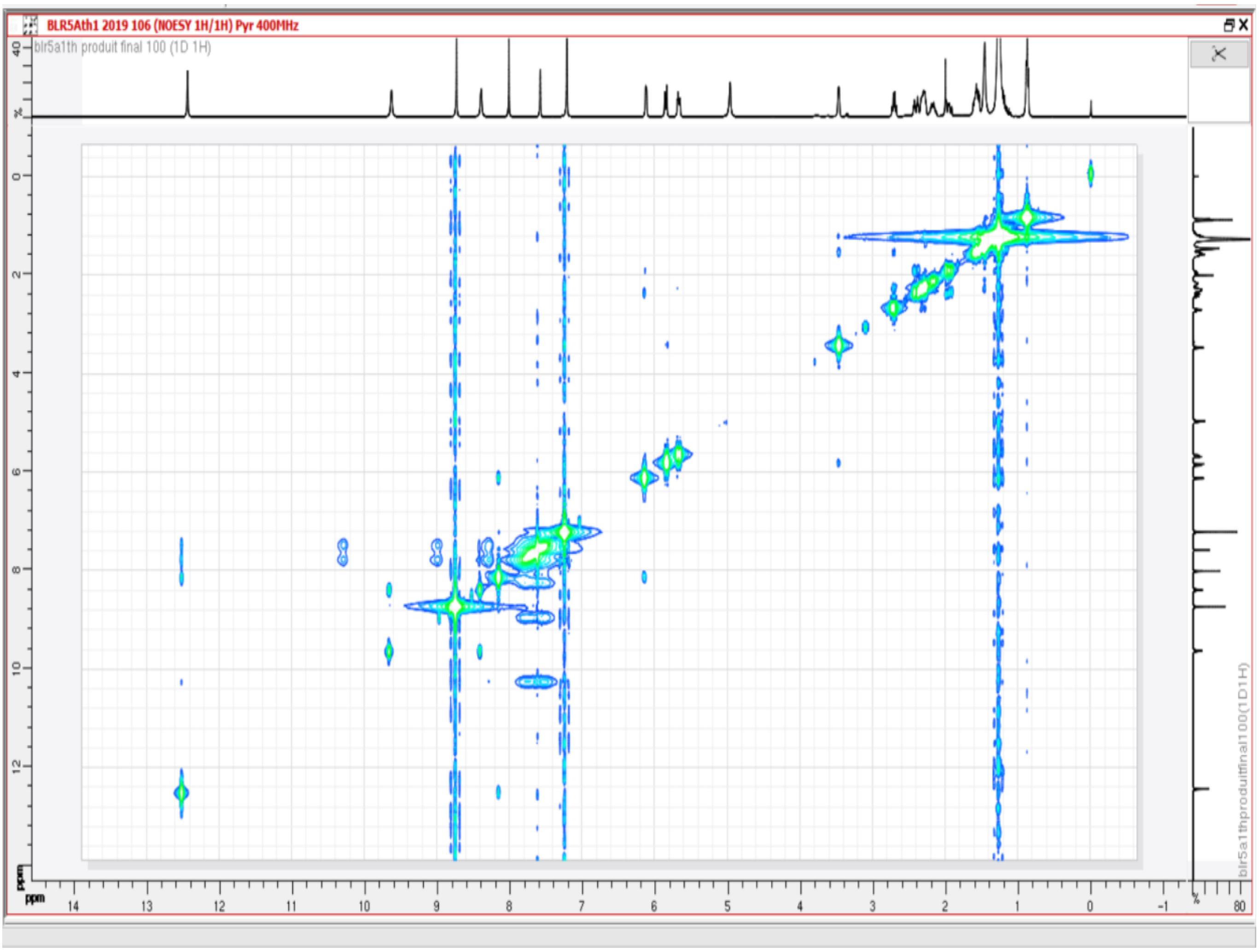

Figure S16: NOESY (Pyridine-d_5, 400 MHz) spectrum of compound **11c**

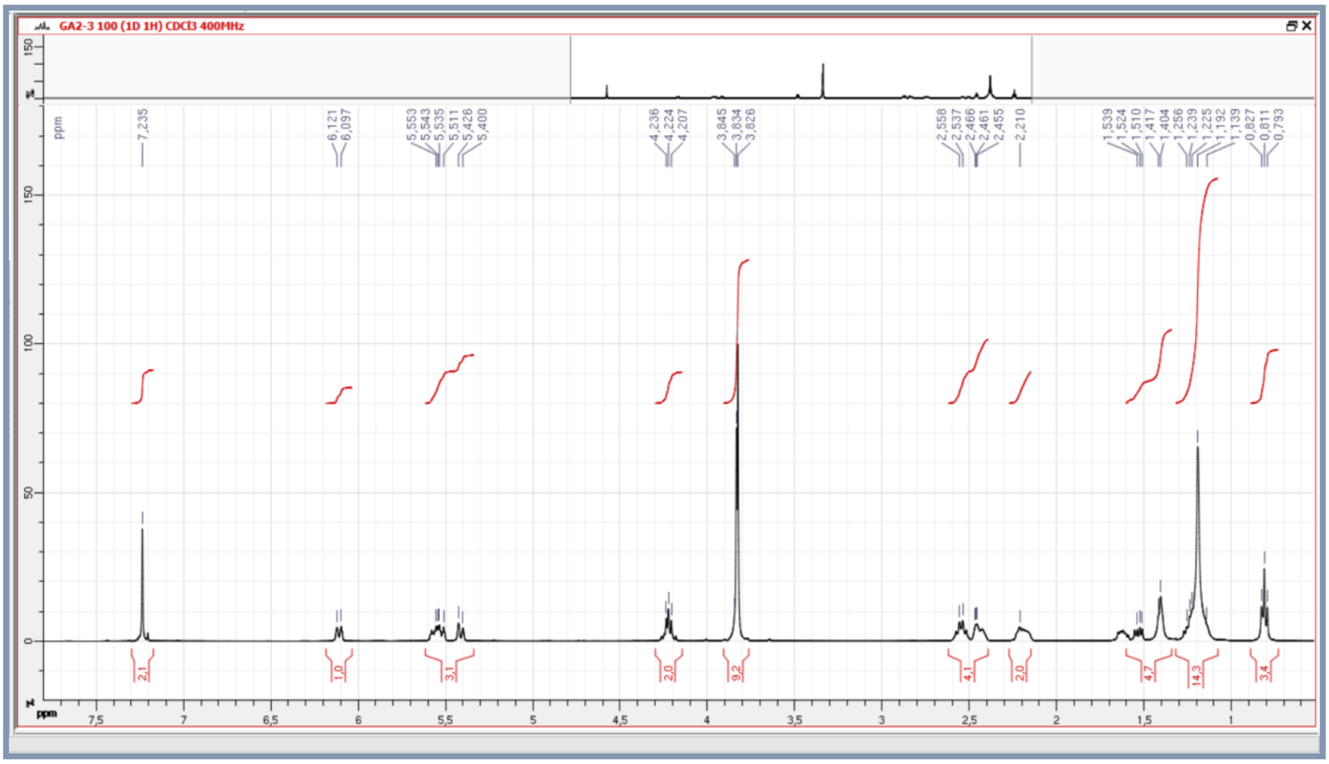

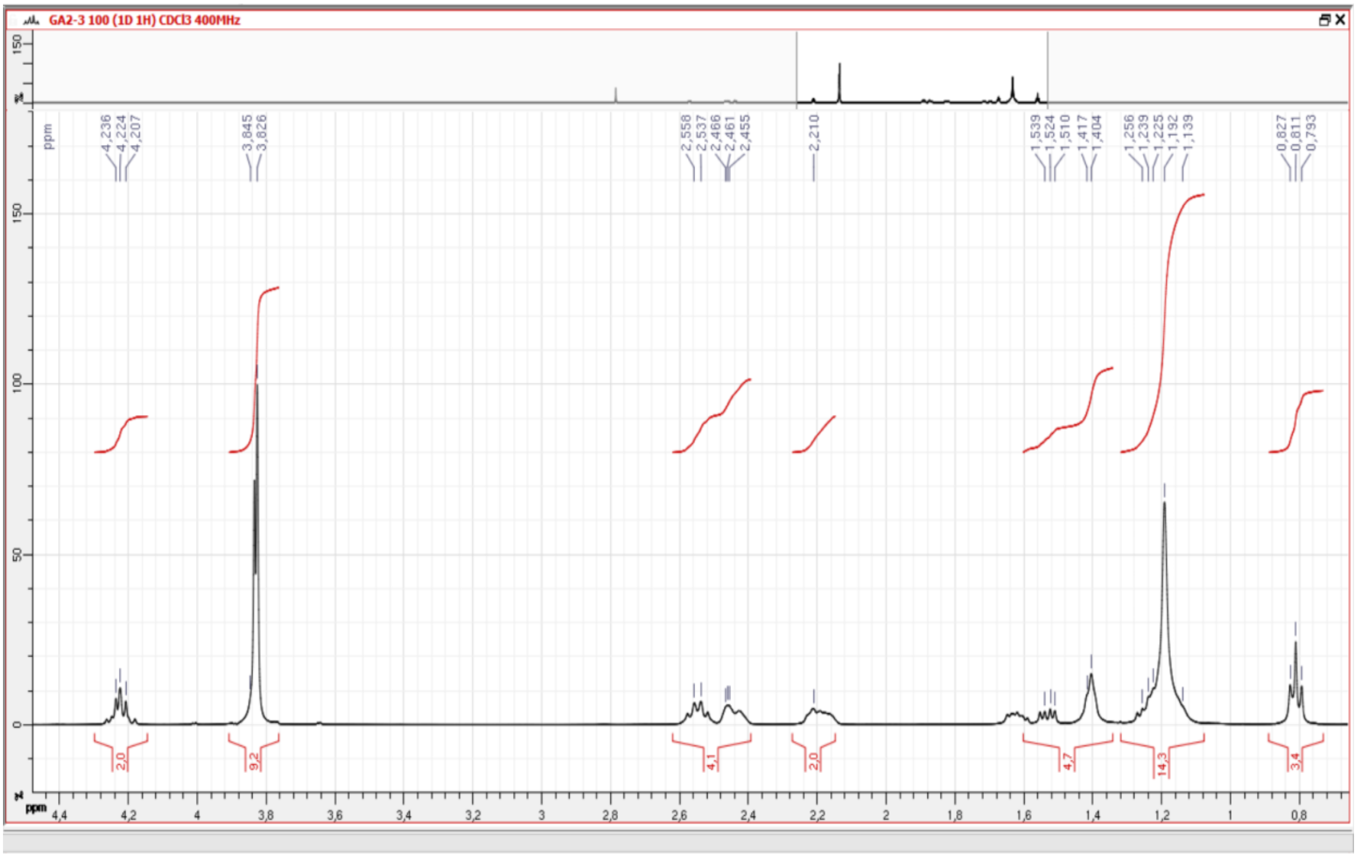

Figure S17: ^{1}H-NMR ($CDCl_3$, 400 MHz) spectrum of compound **11d**

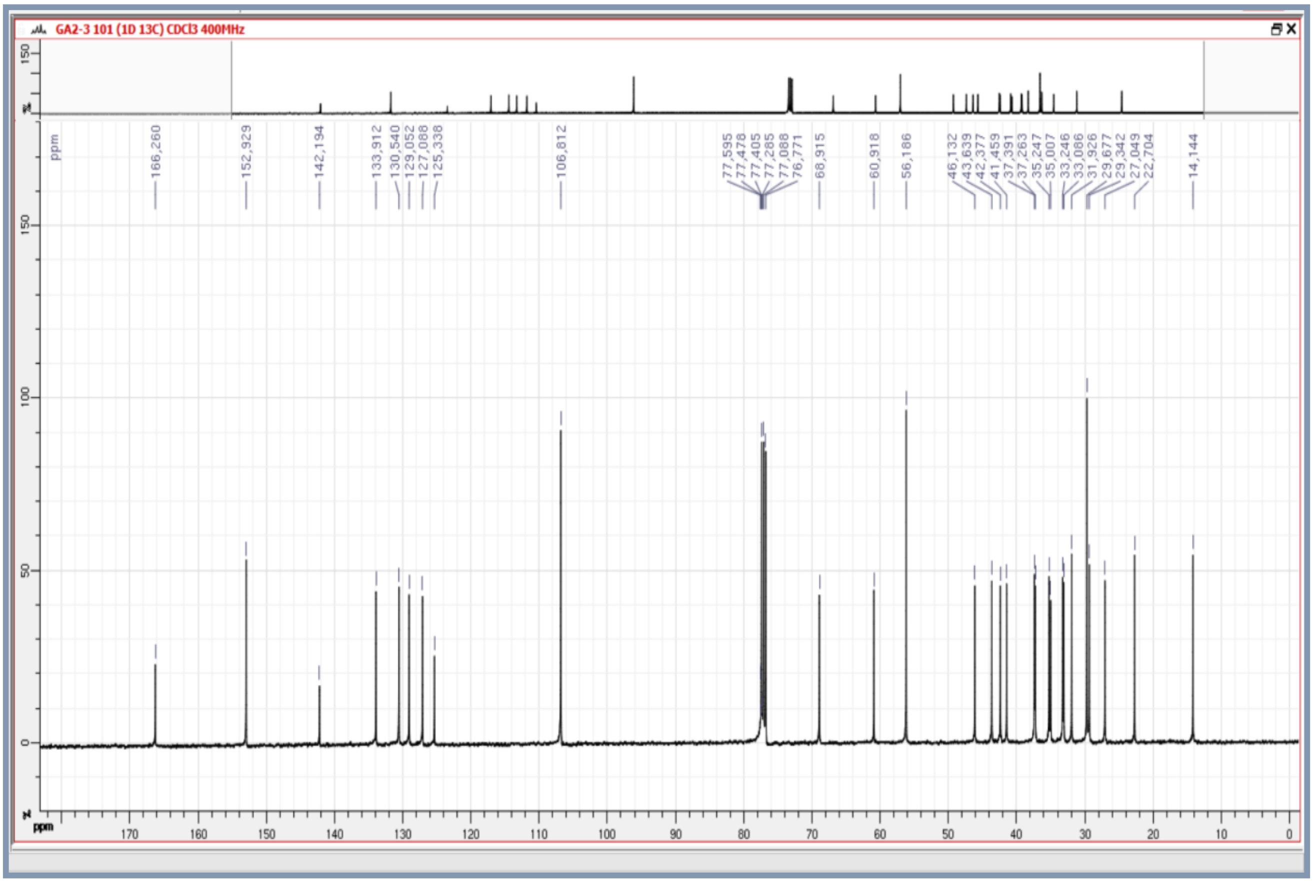

Figure S18: ^{13}C-NMR ($CDCl_3$, 100 MHz) spectrum of compound **11d**

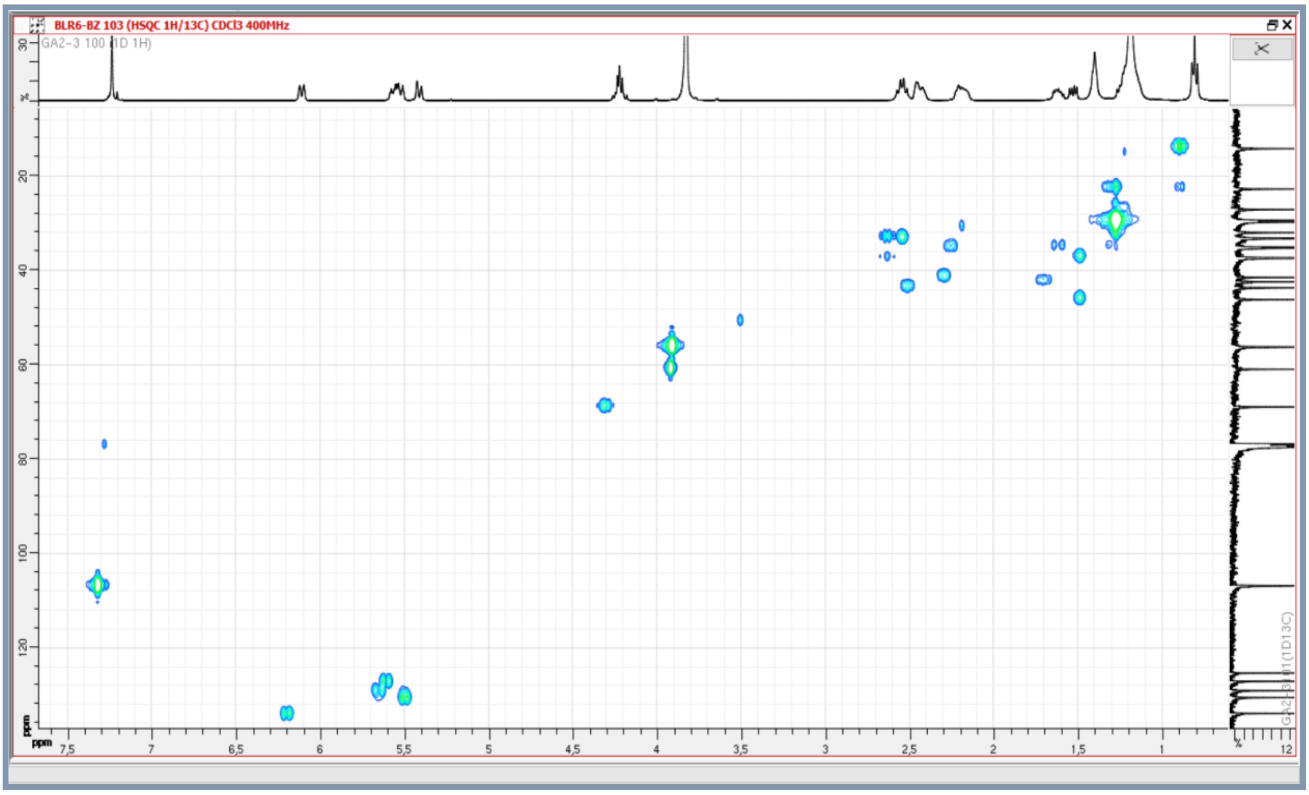

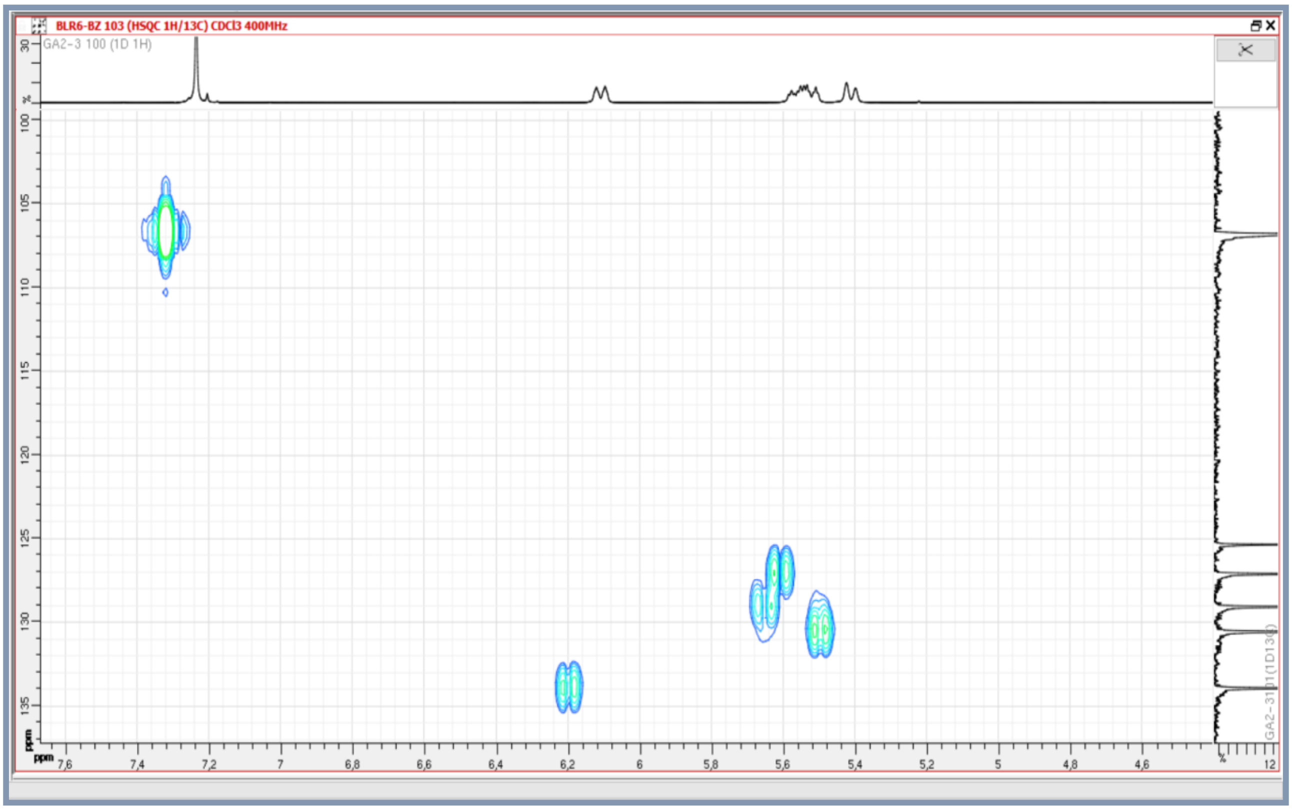

Figure S19: HSQC ($CDCl_3$, 400 MHz) spectrum of compound **11d**

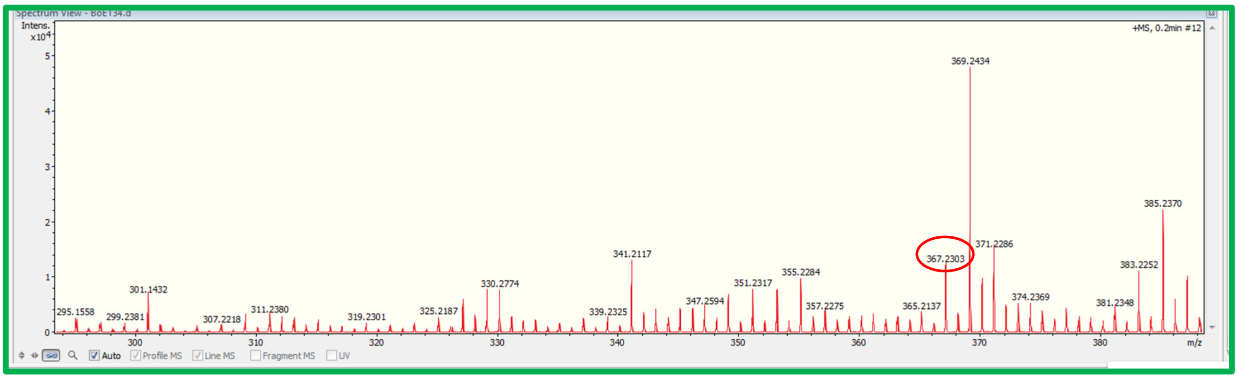

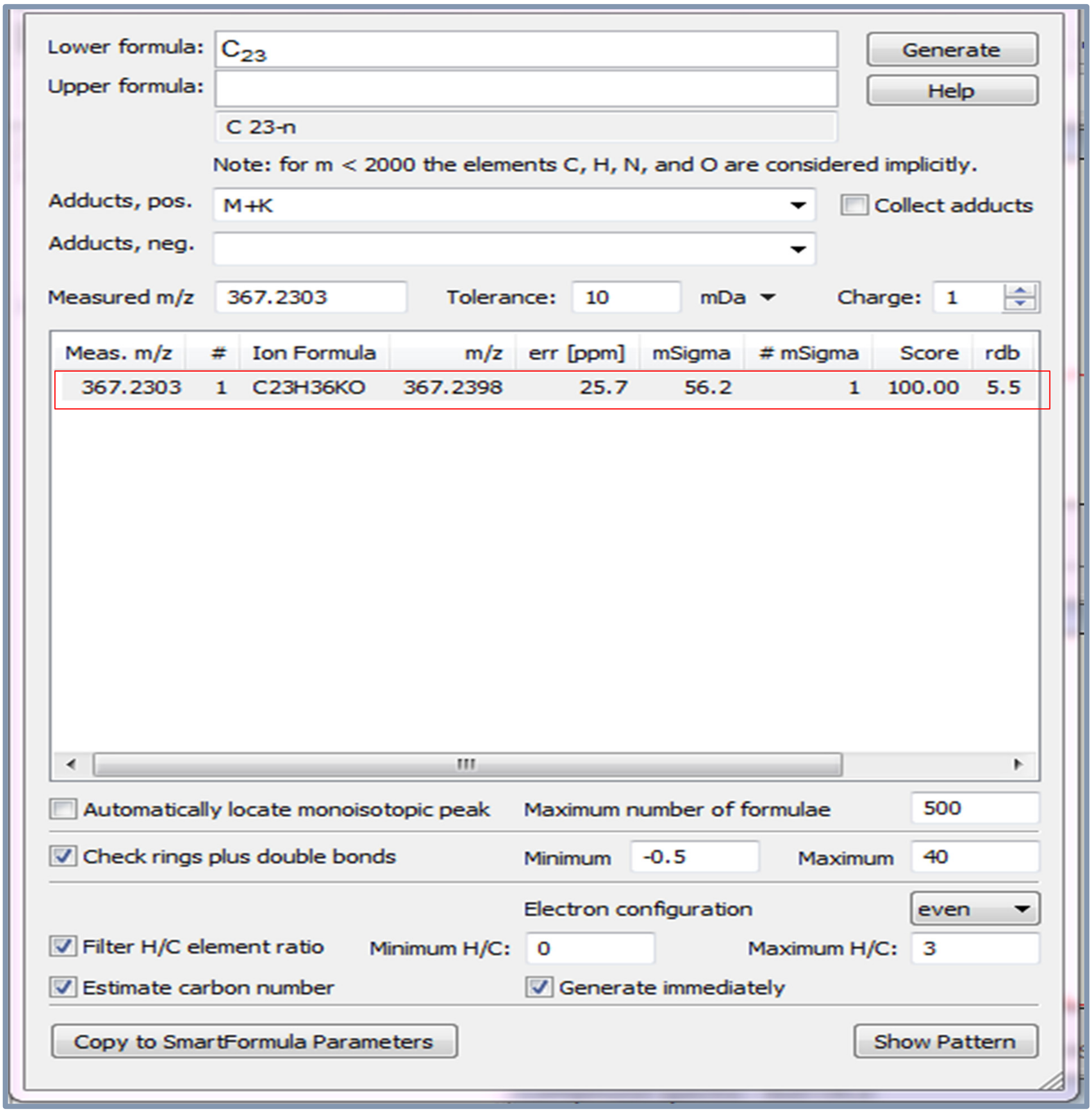

Figure S20: ESI MASS spectrum of the mixture of compound **1** and **2**

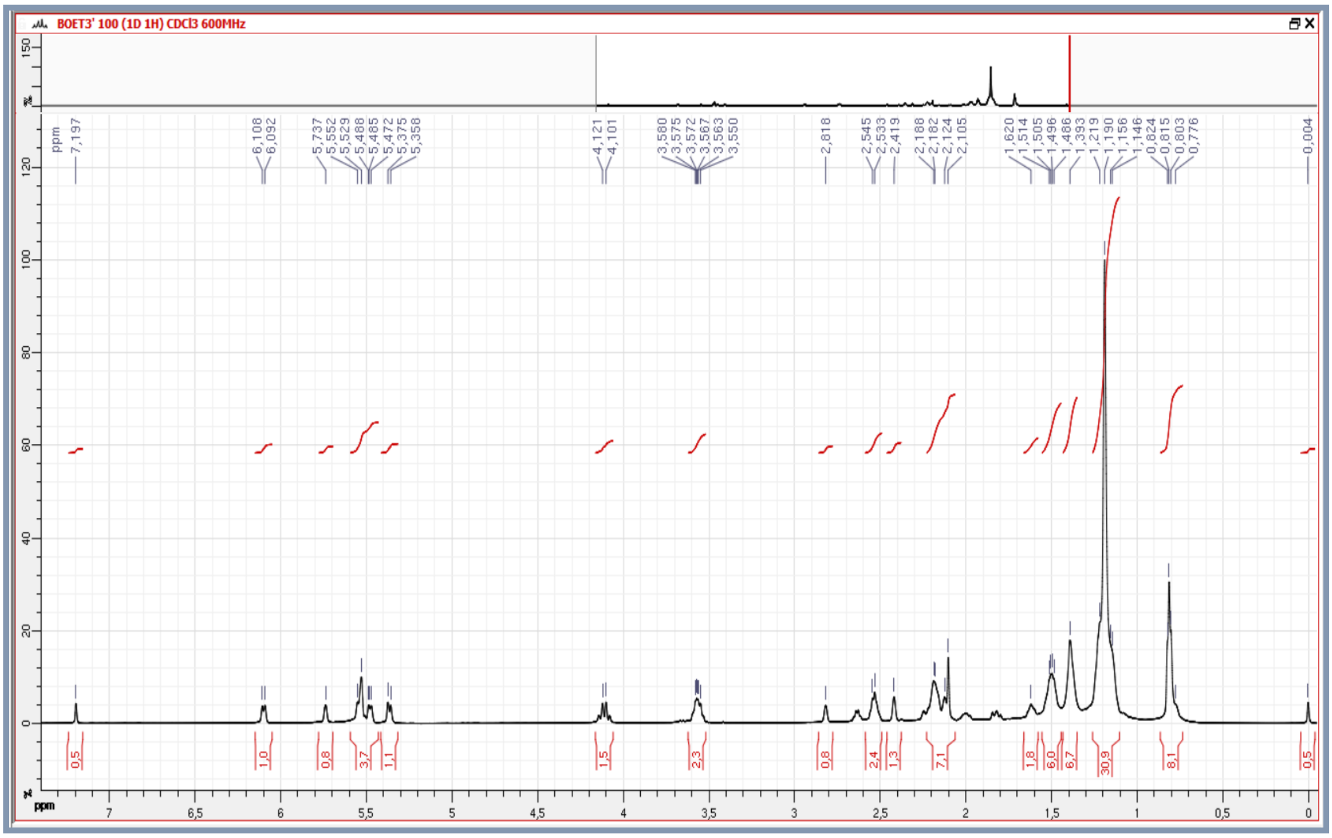
BOET3' 100 (1D 1H) CDCl3 600MHz
7,197
6,108
6,092
5,737
5,552
5,529
5,488
5,485
5,472
5,375
5,358
4,121
4,101
3,580
3,575
3,572
3,567
3,563
3,550
2,818
2,545
2,533
2,419
2,188
2,182
2,124
2,105
1,620
1,514
1,505
1,496
1,486
1,393
1,219
1,190
1,156
1,146
0,824
0,815
0,803
0,776
0,004
0,5
1,0
0,8
3,7
1,1
1,5
2,3
0,8
2,4
1,3
7,1
1,8
6,0
6,7
30,9
8,1
0,5
ppm

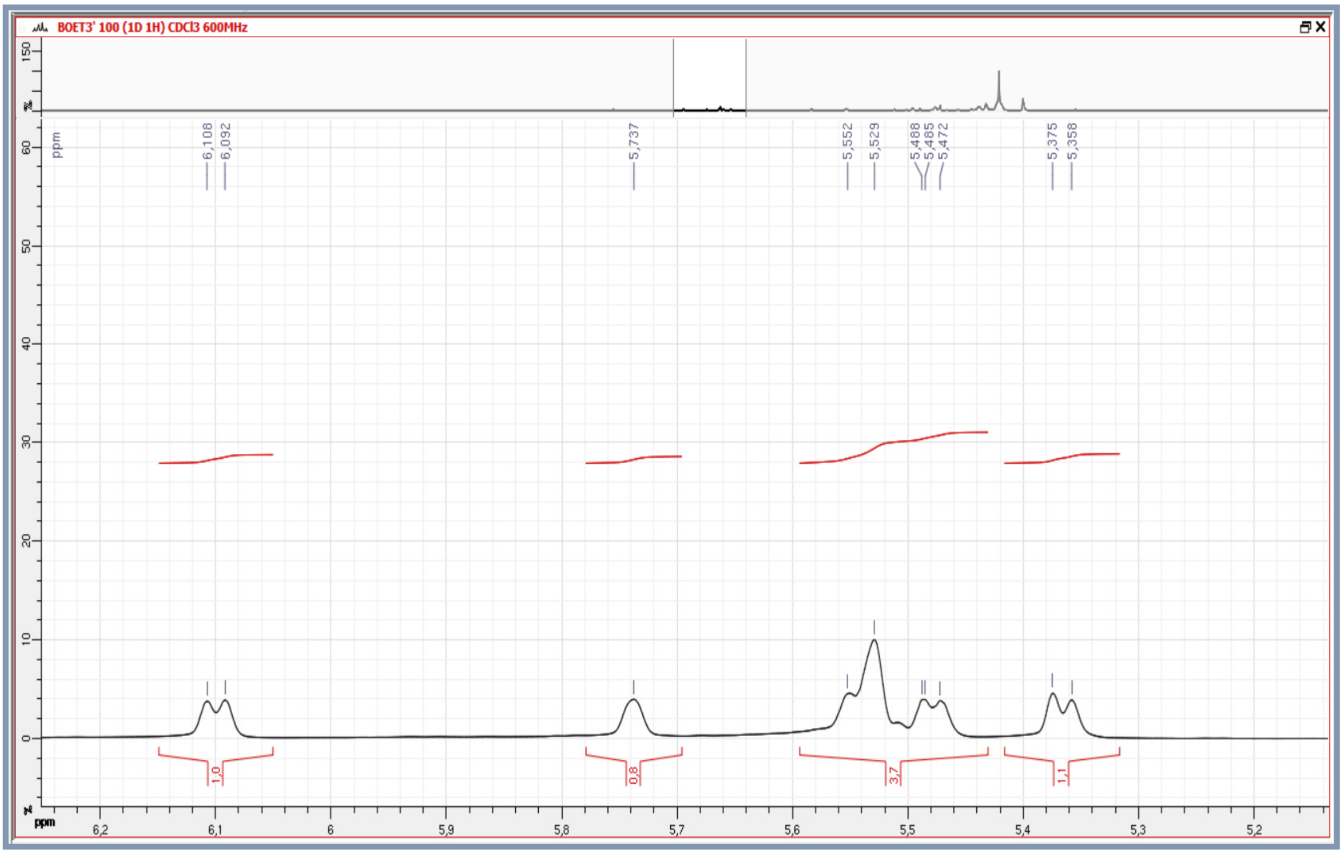
BOET3' 100 (1D 1H) CDCl3 600MHz
6,108
6,092
5,737
5,552
5,529
5,488
5,485
5,472
5,375
5,358
1,0
0,8
3,7
1,1
ppm

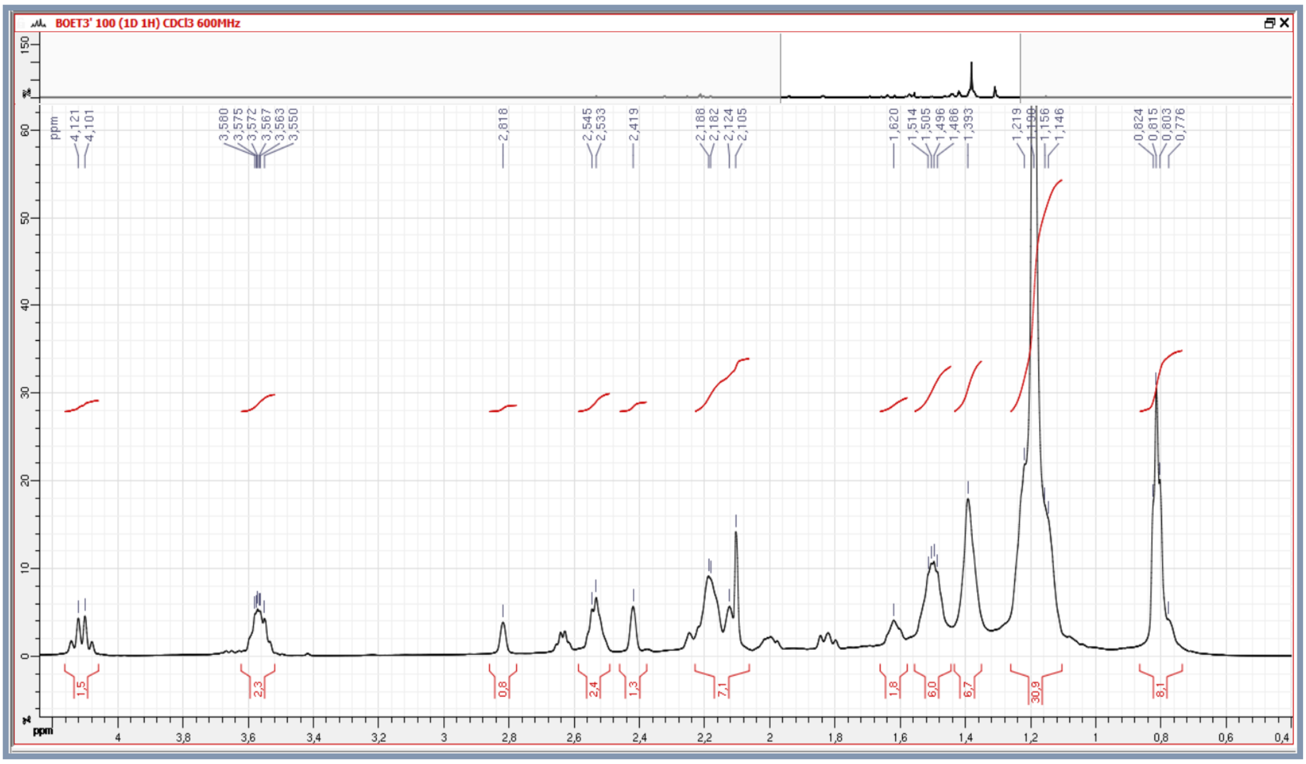

Figure S21: ^{1}H-NMR ($CDCl_3$, 600 MHz) spectrum of the mixture of compound **1** and **2**

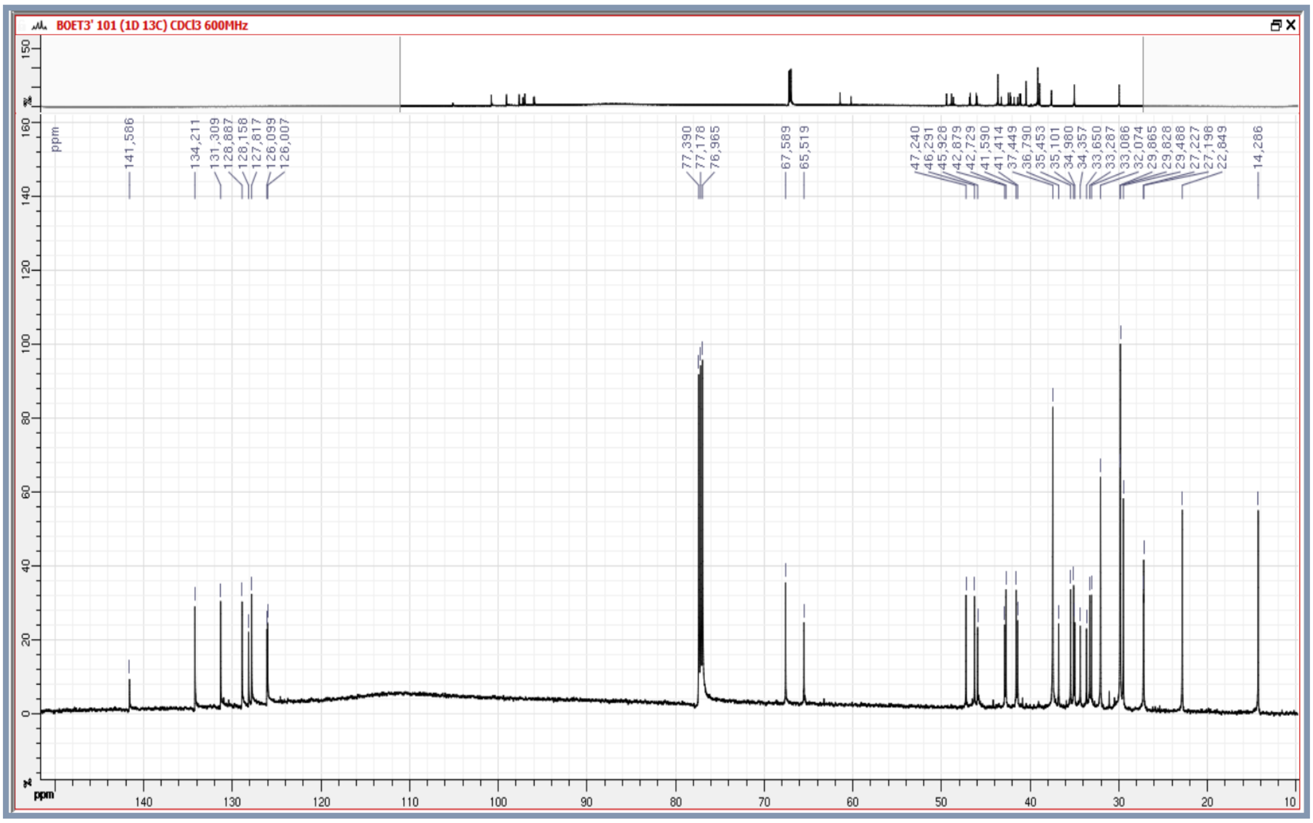

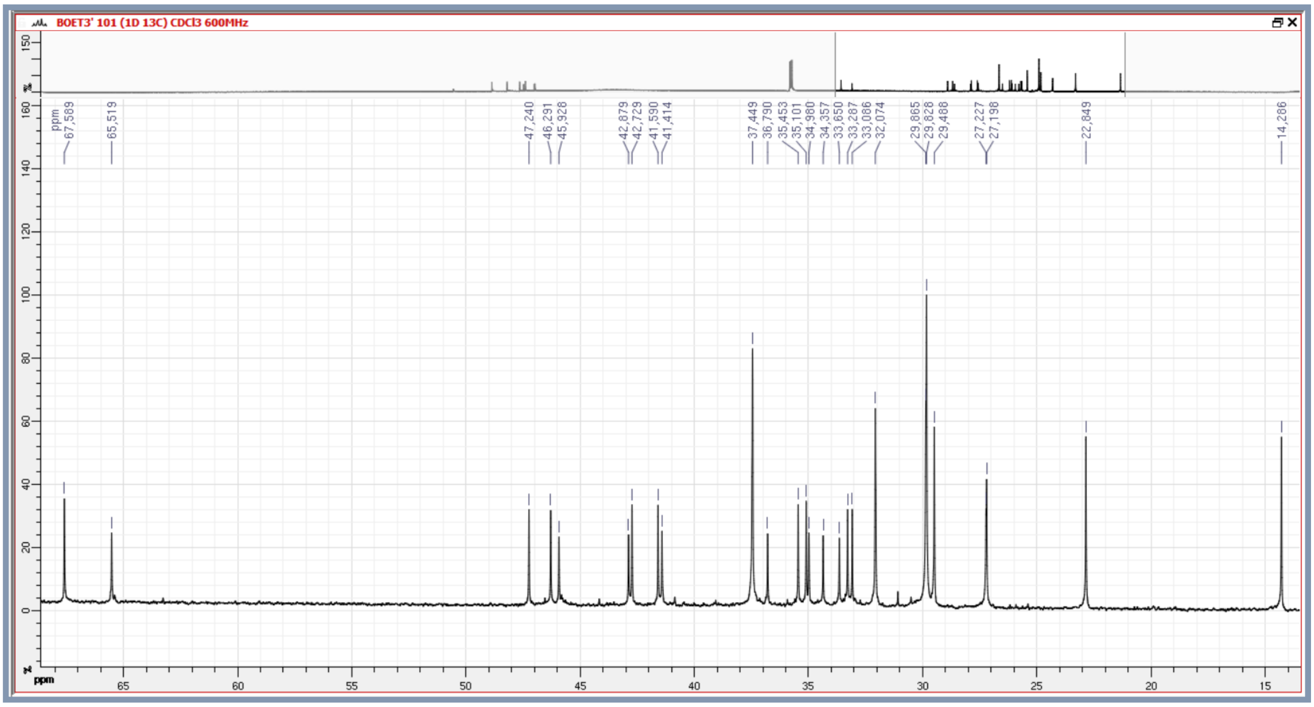

Figure S22:^{13}C-NMR ($CDCl_3$, 150 MHz) spectrum of the mixture of compound **1** and **2**

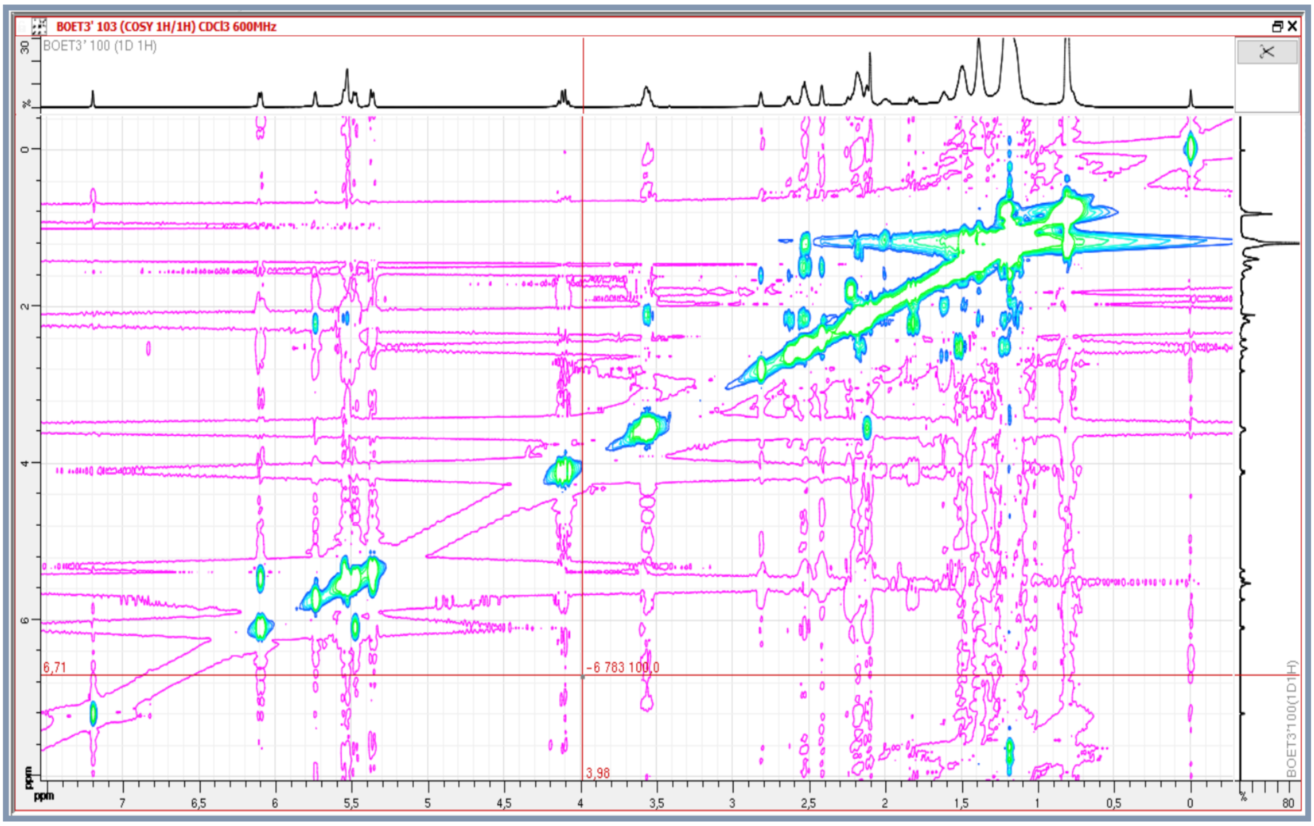

Figure S23: COSY ($CDCl_3$, 600 MHz) spectrum of the mixture of compound **1** and **2**

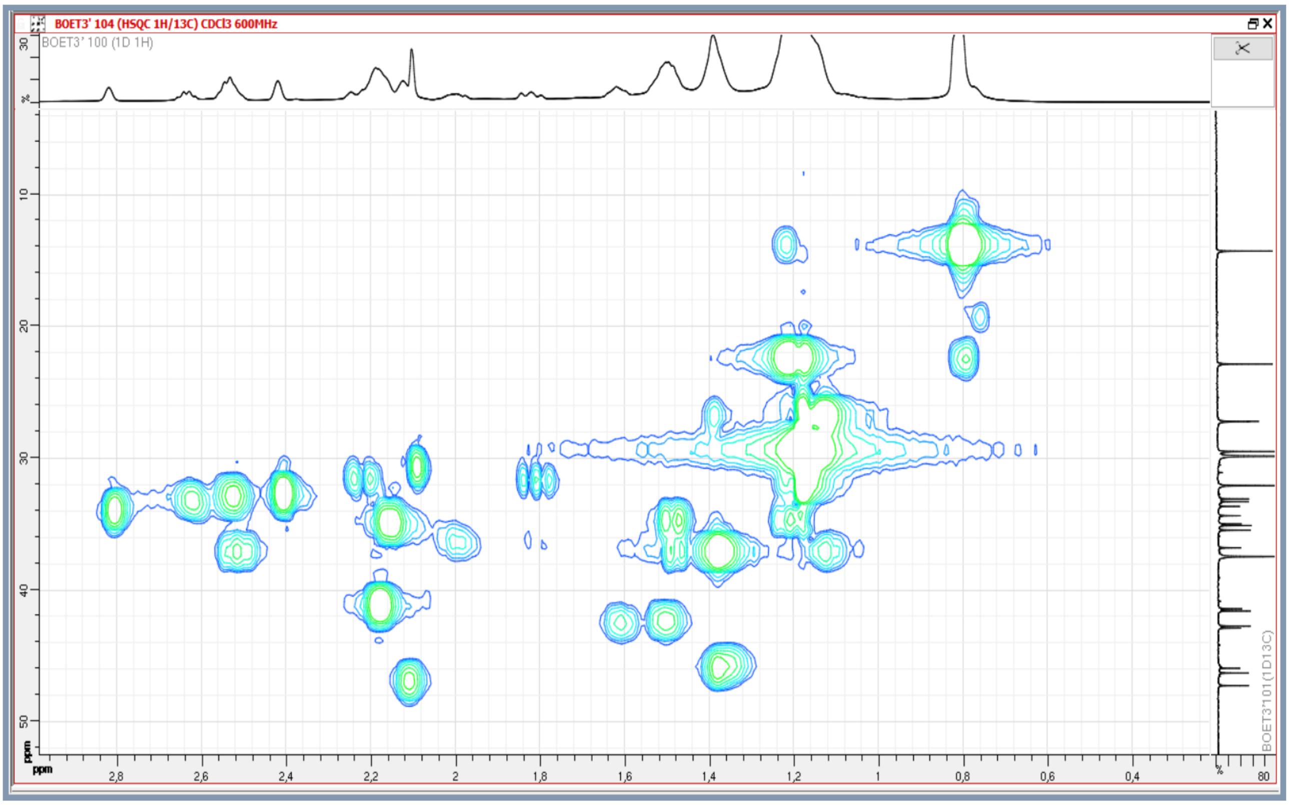

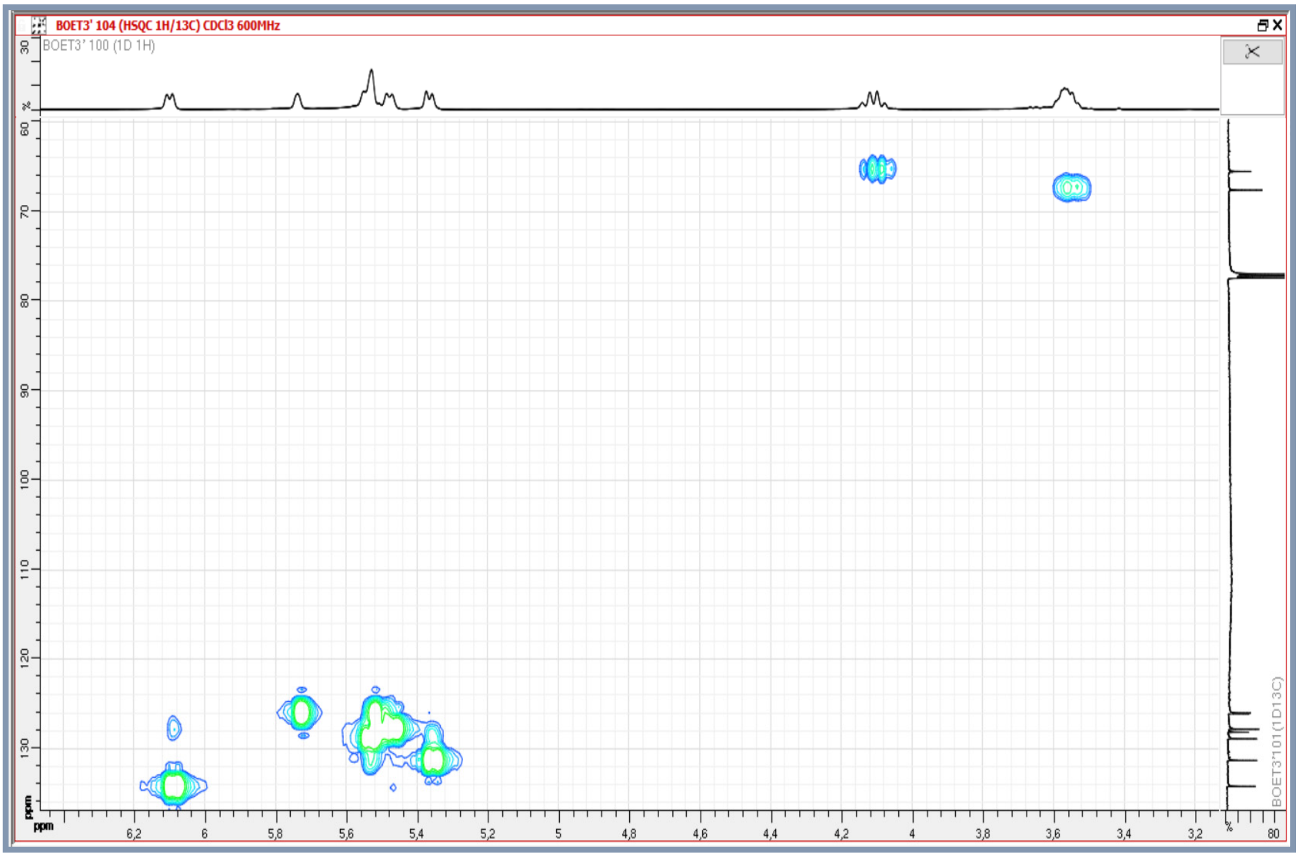

Figure S24: HSQC ($CDCl_3$, 600 MHz) spectrum of the mixture of compound **1** and **2**

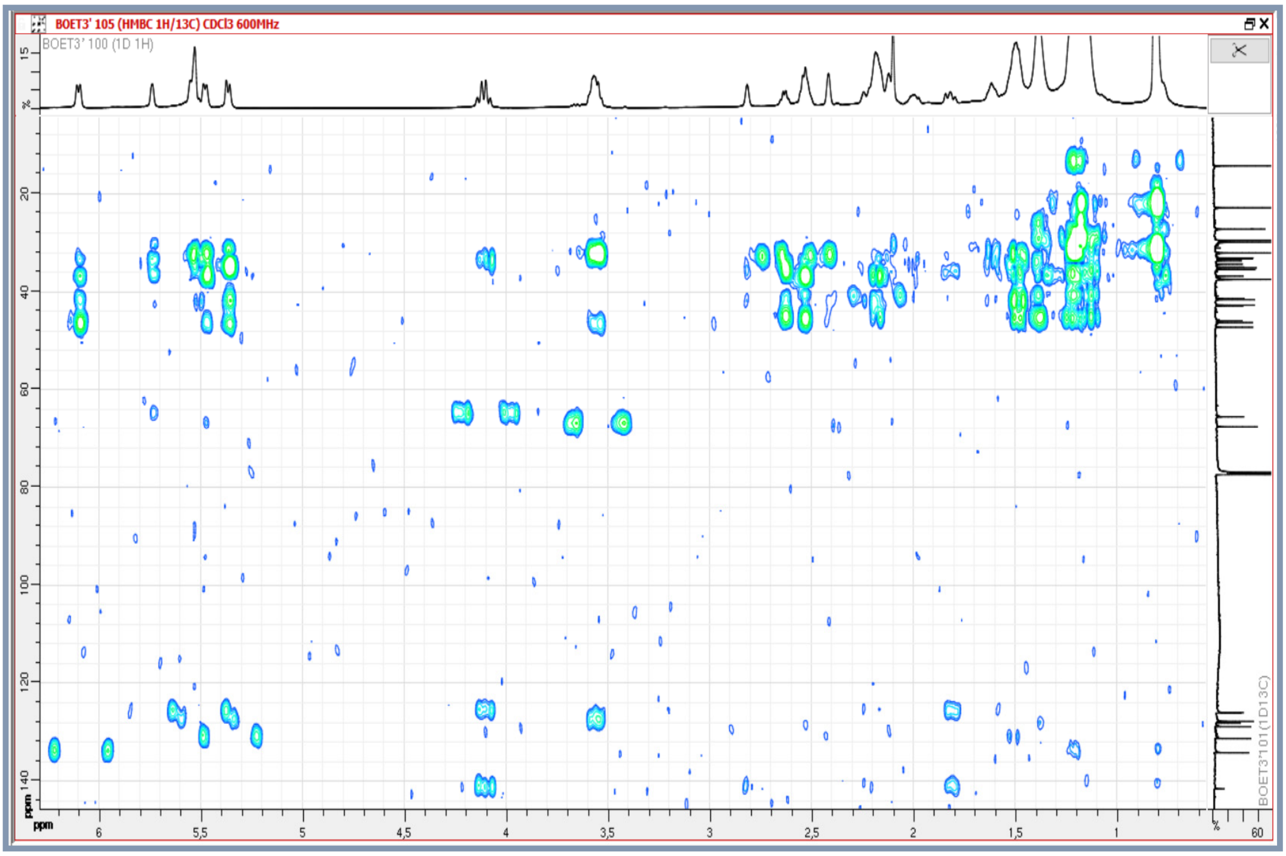
BOET3' 105 (HMBC 1H/13C) CDCl3 600MHz
BOET3' 100 (1D 1H)
BOET3'101(1D13C)
ppm

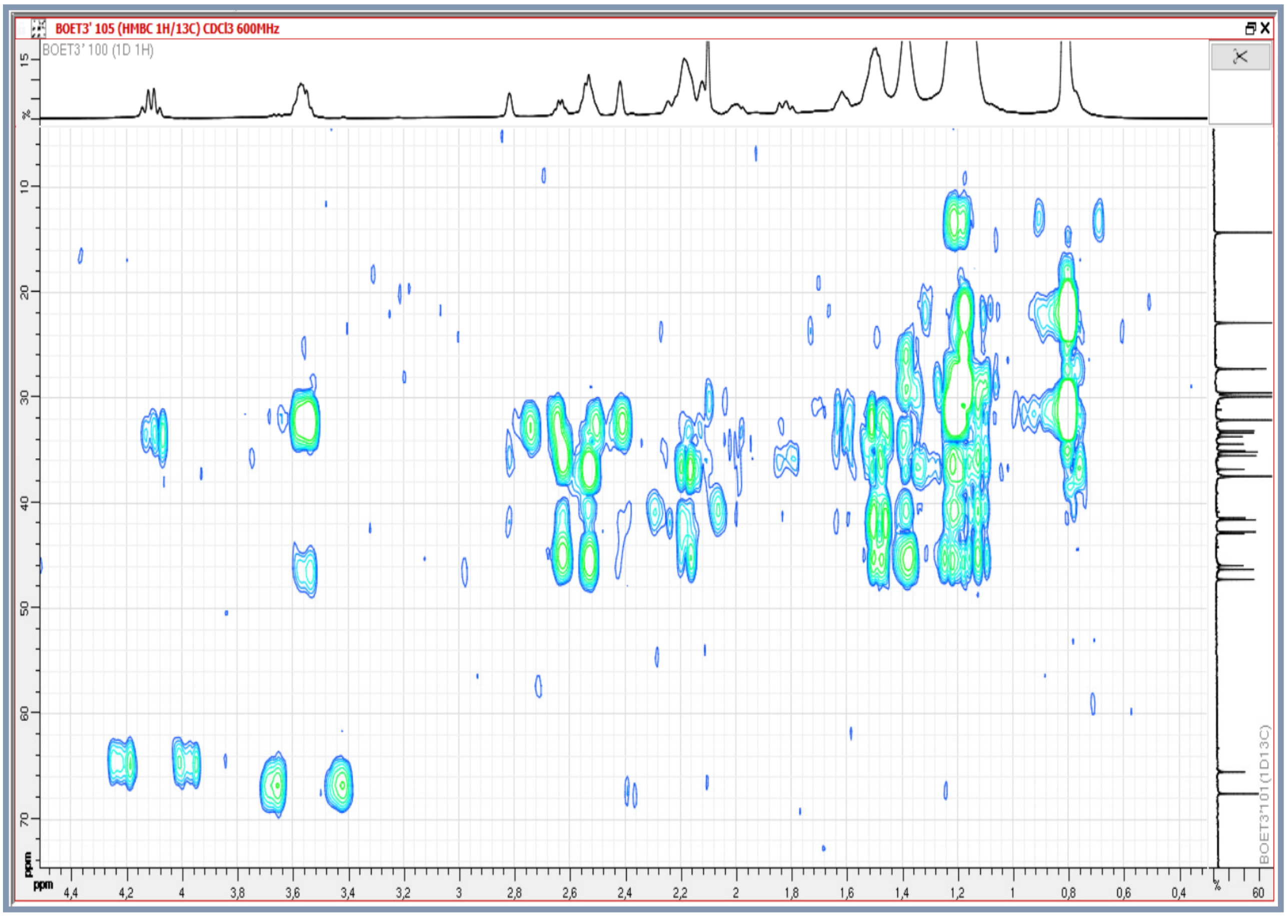
BOET3' 105 (HMBC 1H/13C) CDCl3 600MHz
BOET3' 100 (1D 1H)
BOET3'101(1D13C)
ppm

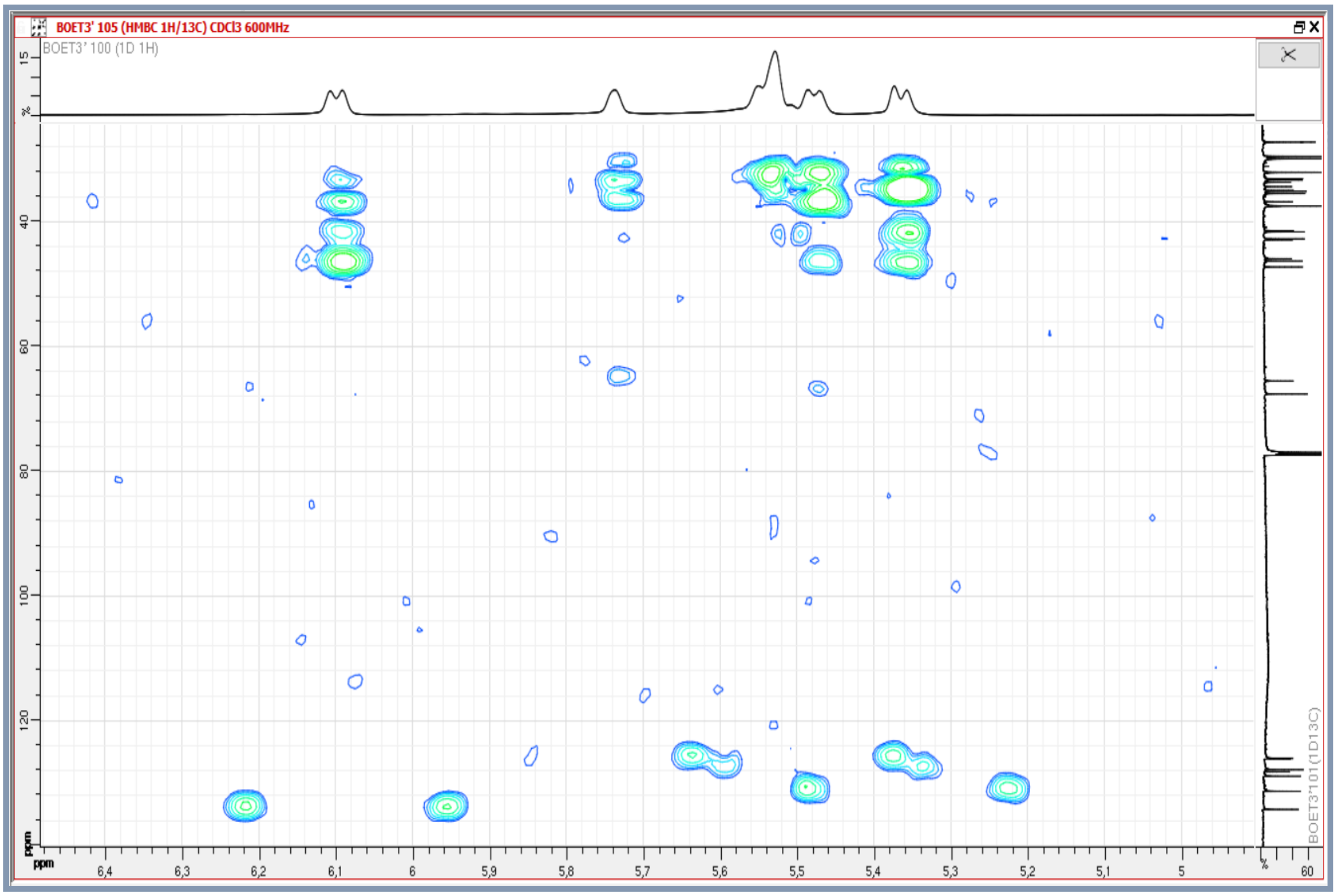

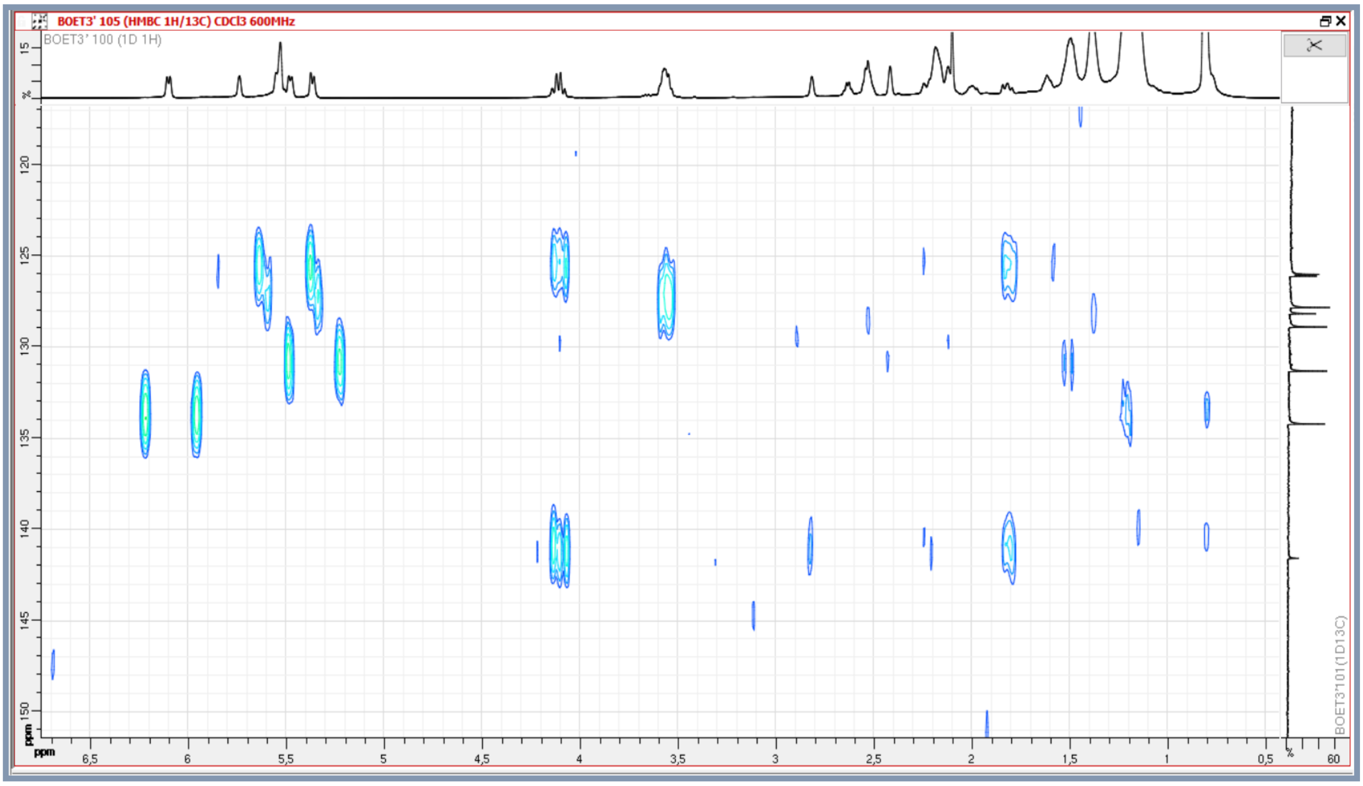

Figure S25: HMBC ($CDCl_3$, 600 MHz) spectrum of the mixture of compound **1** and **2**

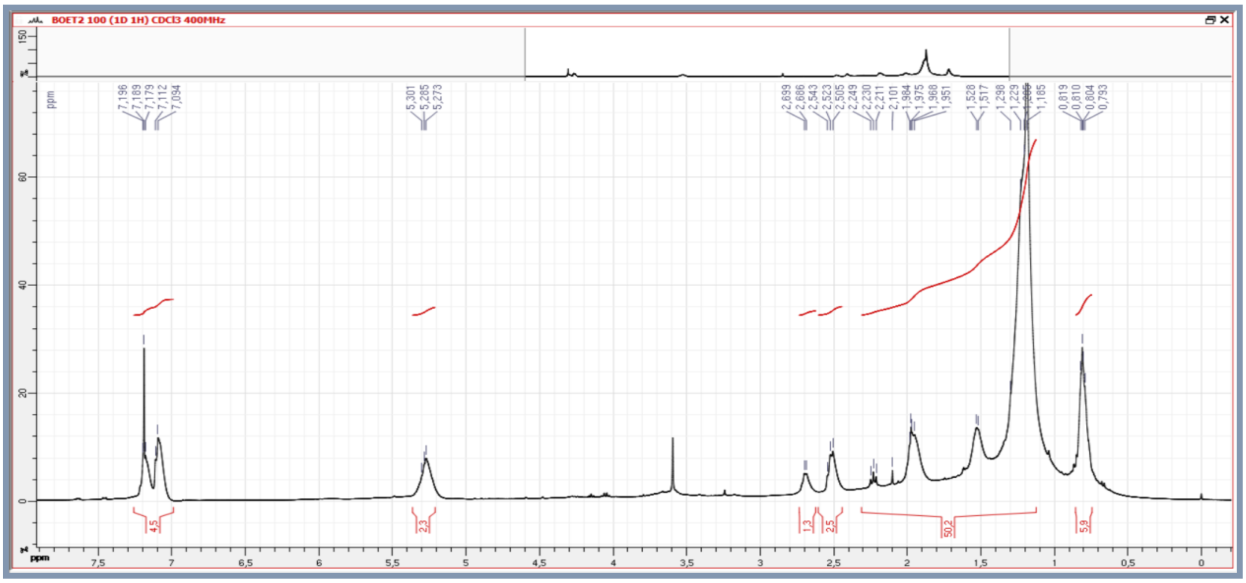

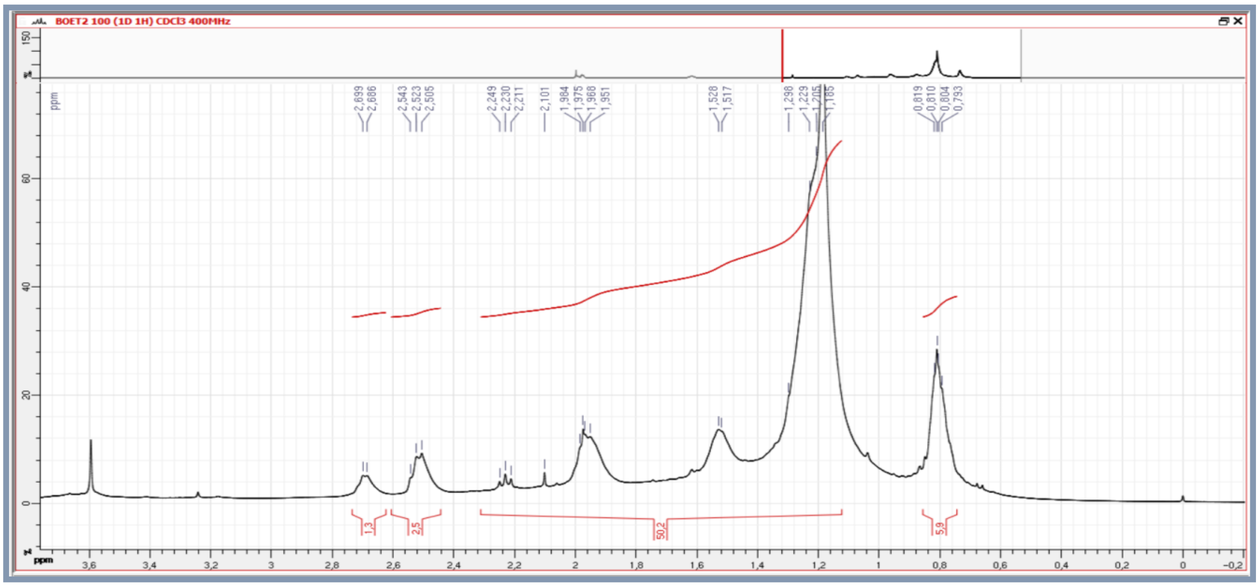

Figure S26: ^{1}H-NMR ($CDCl_3$, 400 MHz) spectrum of compound **3**

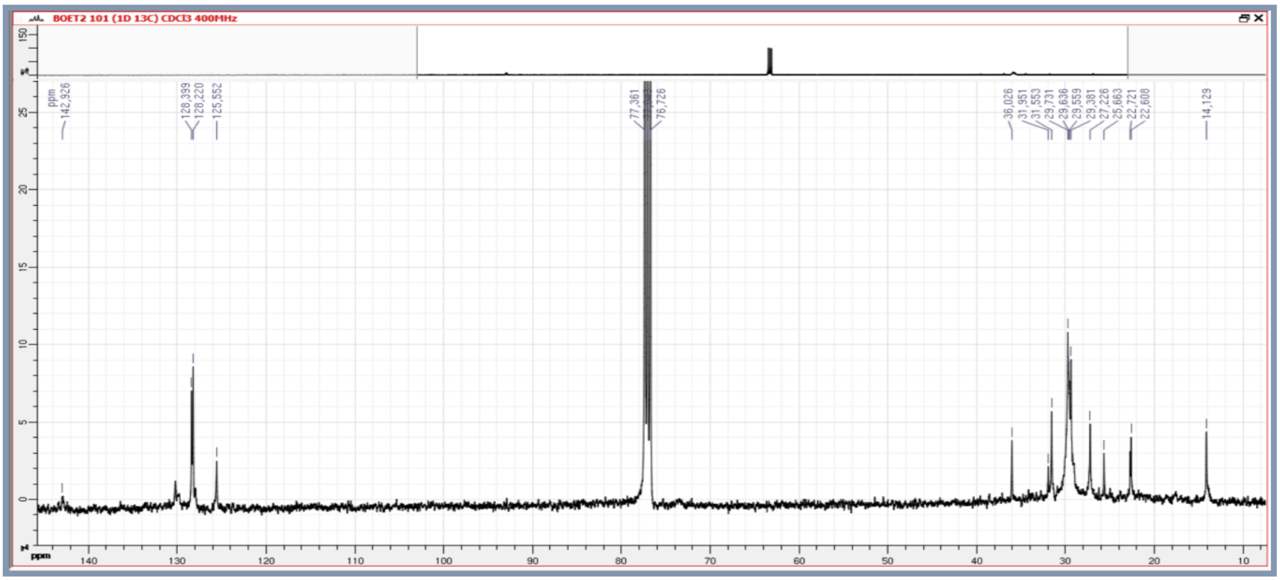

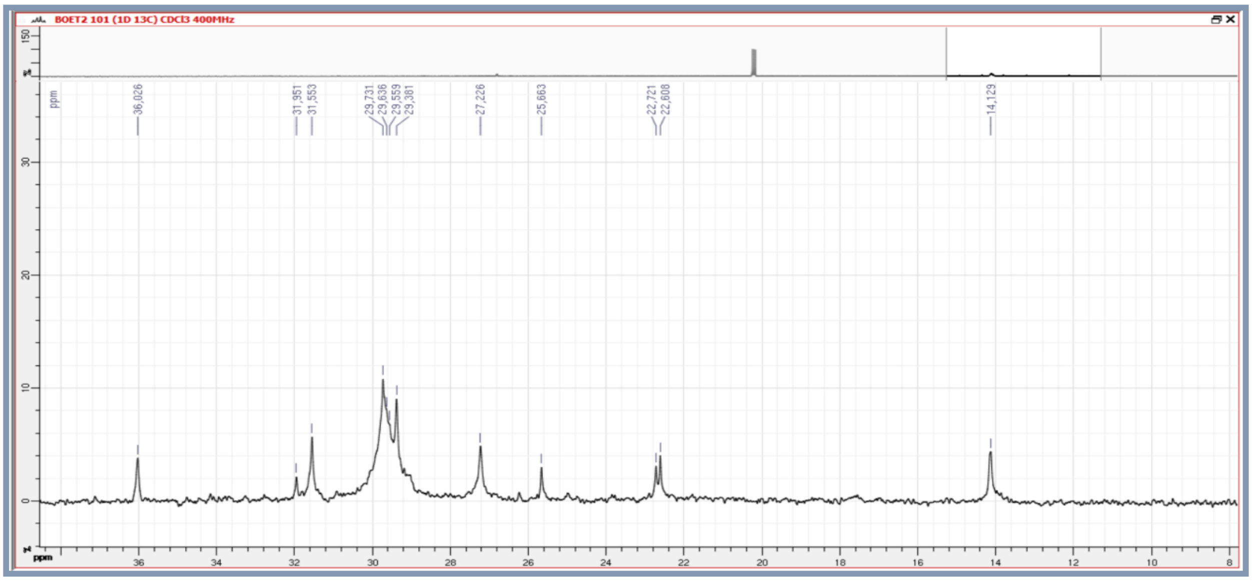

Figure S27: ^{13}C-NMR ($CDCl_3$, 100 MHz) spectrum of compound **3**

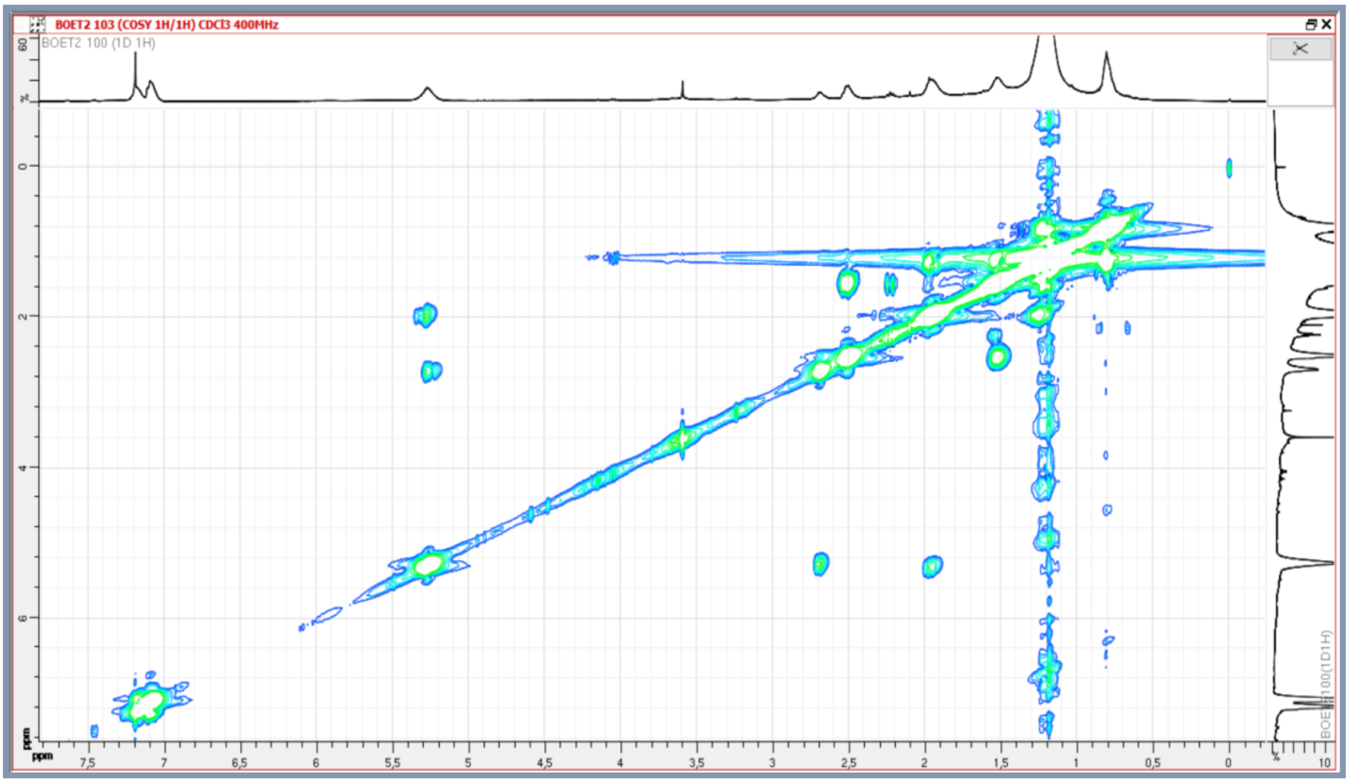

Figure S28: COSY ($CDCl_3$, 400 MHz) spectrum of compound **3**

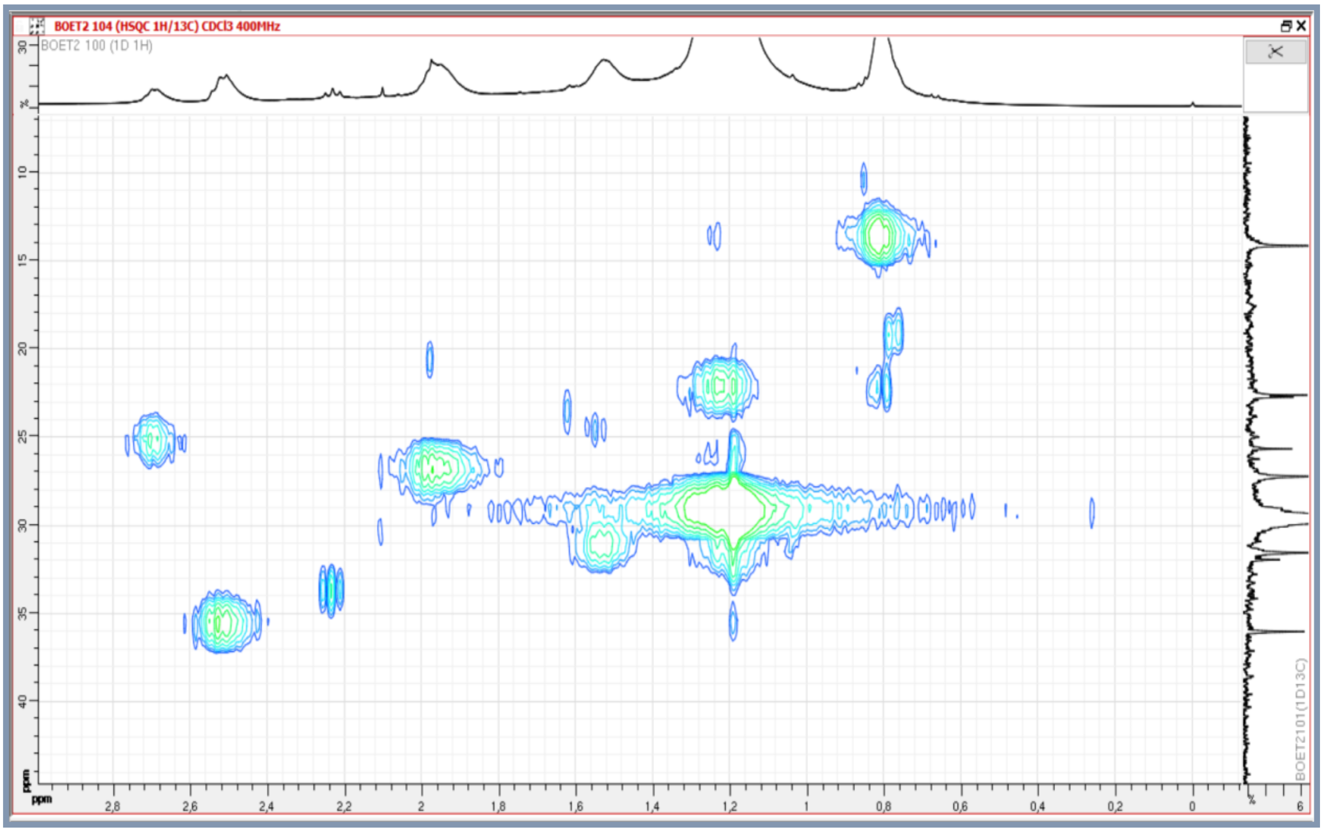

Figure S29: HSQC ($CDCl_3$, 400 MHz) spectrum of compound **3**

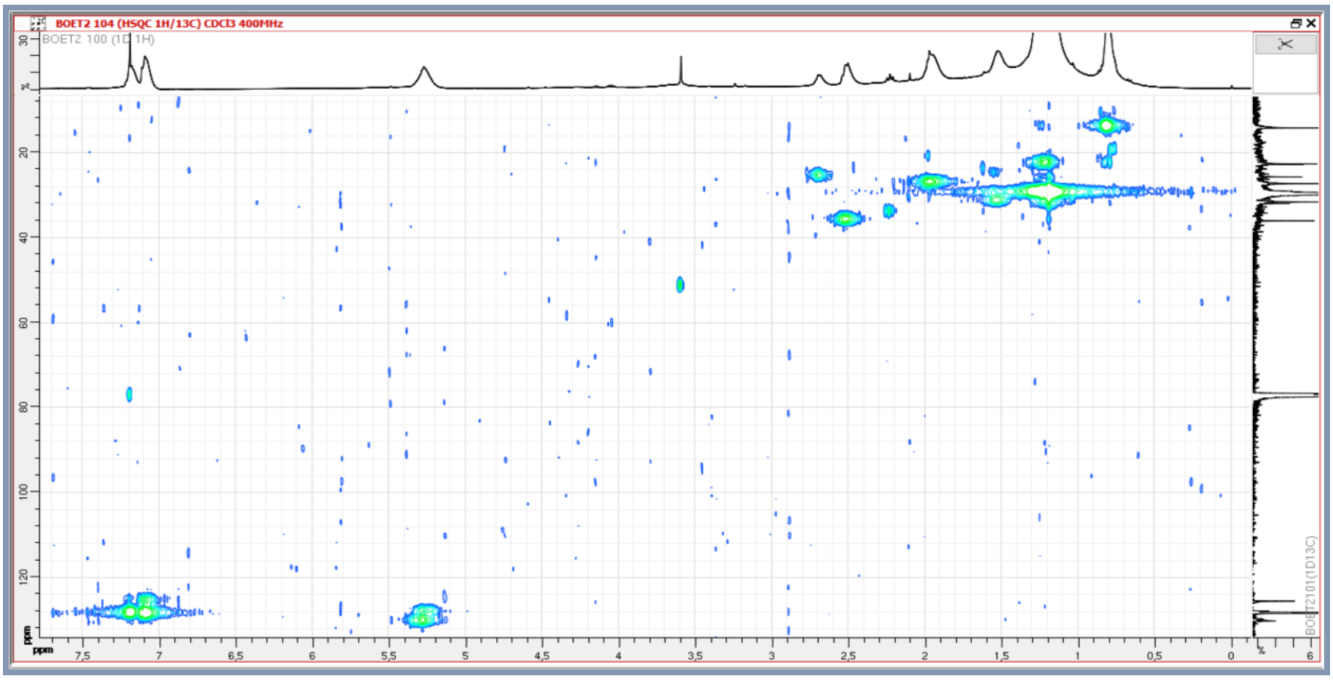

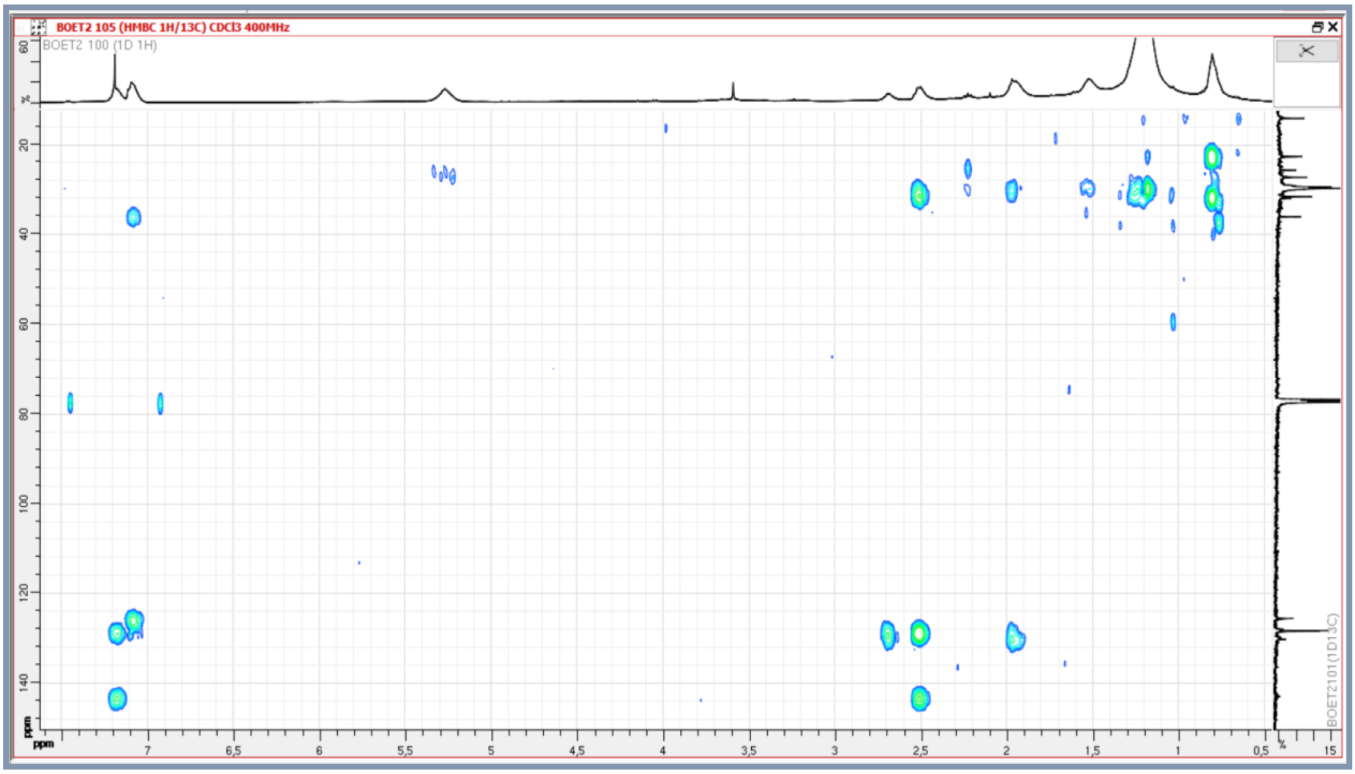

Figure S30: HMBC ($CDCl_3$, 400 MHz) spectrum of compound **3**

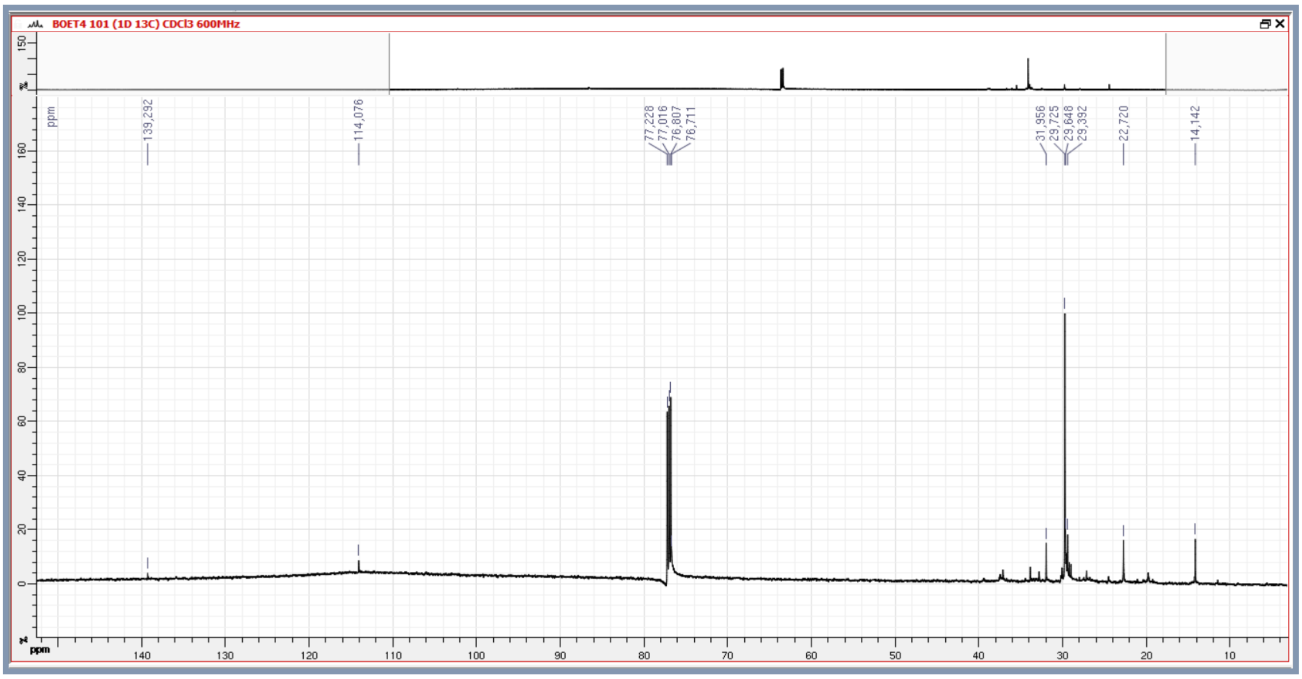

Figure S31: ^{13}C-NMR ($CDCl_3$, 150 MHz) spectrum of compound **4**

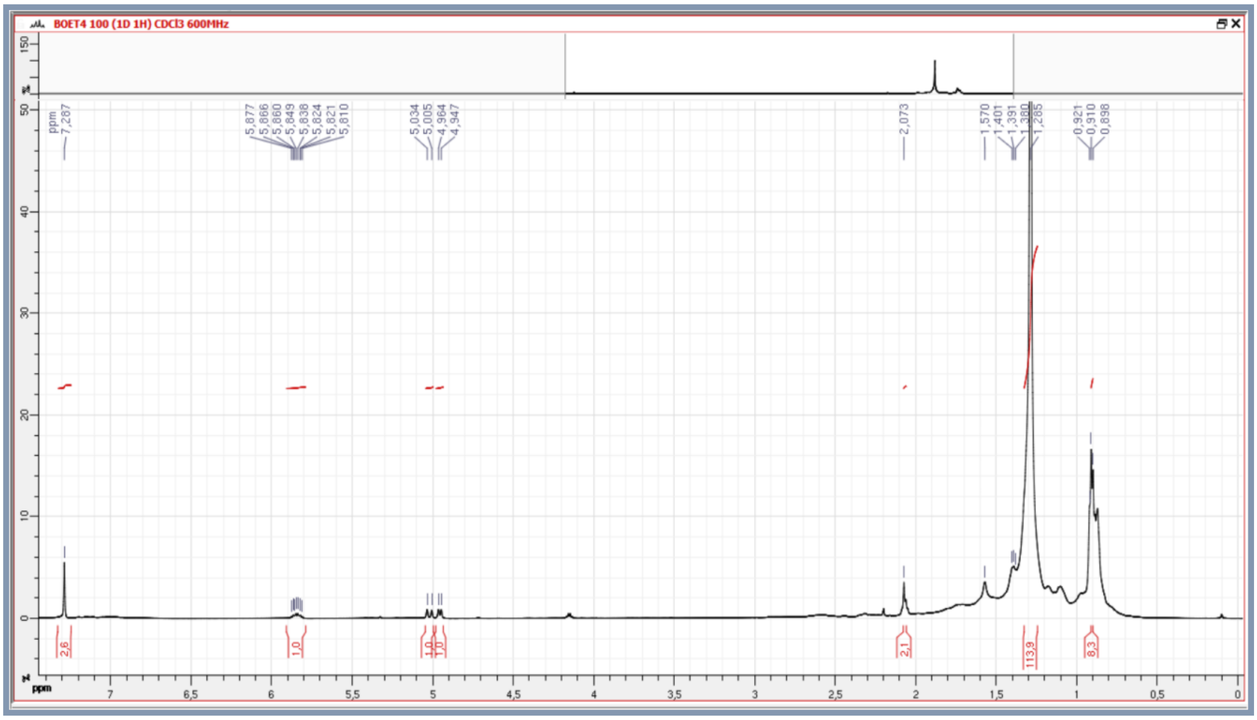

Figure S32: ^{1}H-NMR ($CDCl_3$, 600 MHz) spectrum of compound **4**

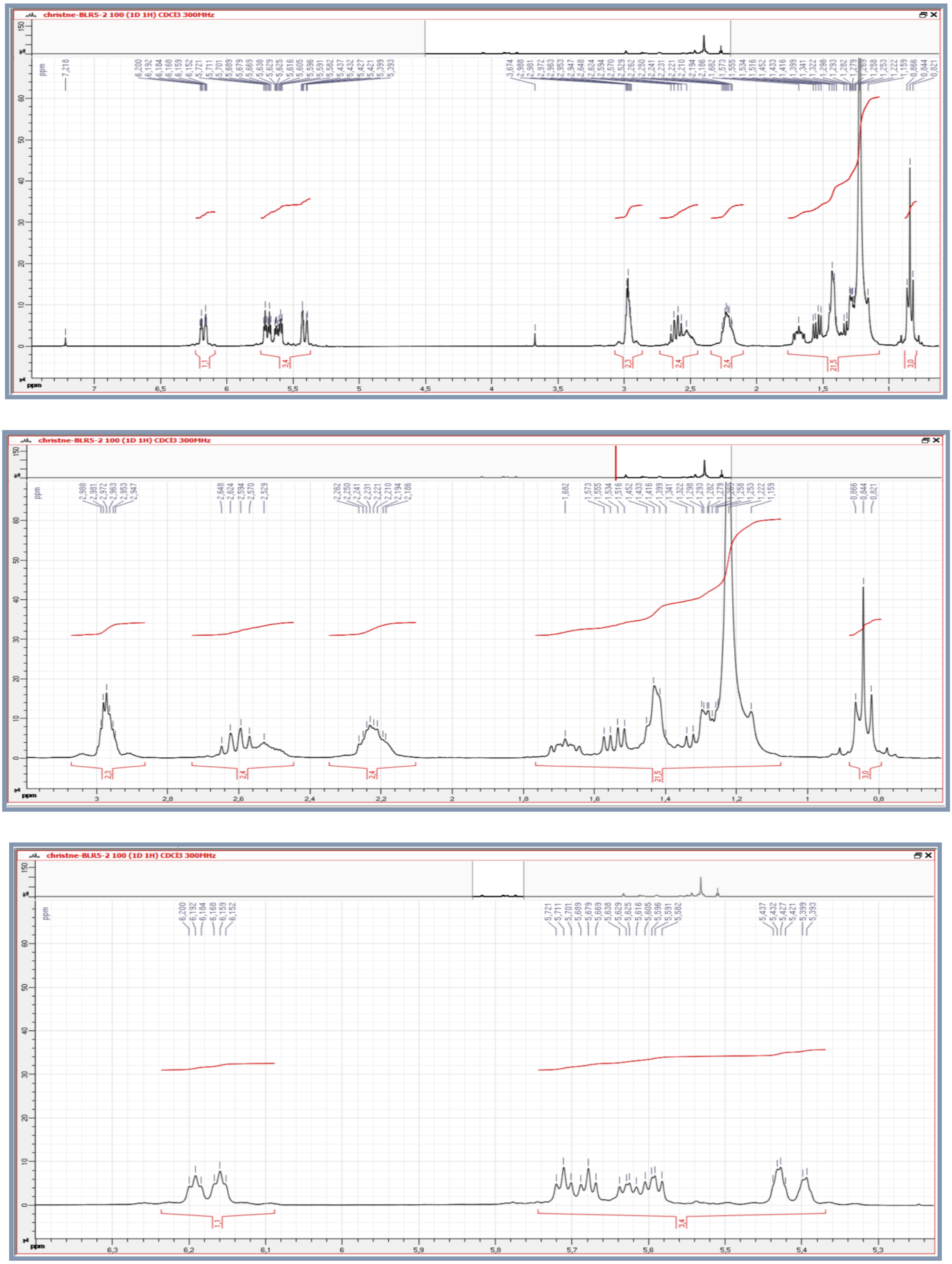

Figure S33: ^{1}H-NMR ($CDCl_3$, 300 MHz) spectrum of compound **11**

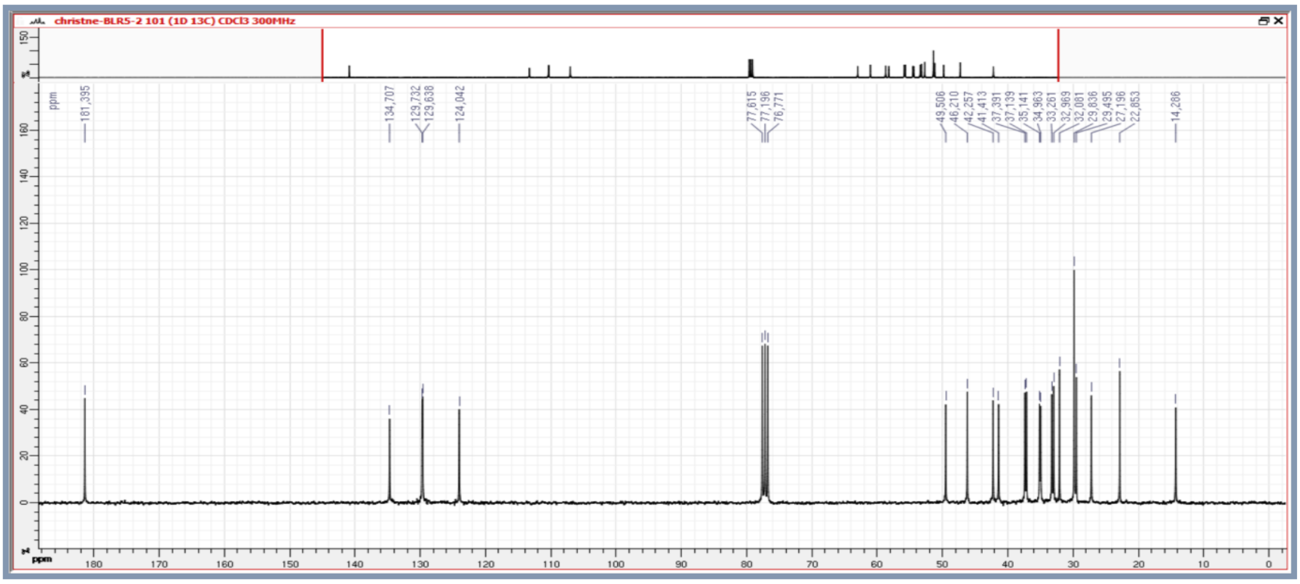

Figure S34: ^{13}C-NMR ($CDCl_3$, 75 MHz) spectrum of compound **11**
